# Therapeutic efficacy of genetically engineered neural stem cells in cerebral ischemia: a systematic review and meta-analysis

**DOI:** 10.1186/s12967-025-07603-y

**Published:** 2026-02-19

**Authors:** Sandra Li, Ricki Anne Solis Marzan, Chuanyu Wei, Abdul Razak, Connie H. Y. Wong, Justin Moore, Courtney A. McDonald

**Affiliations:** 1 Department of Surgery, Faculty of Medicine, Nursing and Health Sciences, Monash University, Melbourne, Australia; 2 The Ritchie Centre, Hudson Institute of Medical Research, Melbourne, Australia; 3 Centre for Inflammatory Diseases, Department of Medicine, School of Clinical Sciences at Monash Health, Monash University, Melbourne, Australia; 4 Department of Neurosurgery, Monash Medical Centre, Monash Health, Melbourne, Australia; 5 Department of Obstetrics and Gynaecology, Faculty of Medicine, Nursing and Health Sciences, Monash University, 27-31 Wright St, Clayton, Melbourne, VIC 3168 Australia; 6 Department of Paediatrics, Faculty of Medicine, Nursing and Health Sciences, Monash University, Melbourne, Australia

**Keywords:** Cerebral ischemia, Stroke, Neural stem cells, Genetic engineering, Regeneration, Regenerative medicine

## Abstract

**Background:**

Stroke is one of the most common causes of death and permanent neurological disabilities worldwide yet neuroprotective or regenerative therapies do not exist. Genetically modified neural stem cells (NSC) could help overcome key limitations of naïve or unmodified NSCs and improve therapeutic efficacy. The aim of this systematic review and meta-analysis is to evaluate existing preclinical literature using animal models to compare the therapeutic effects of genetically modified NSCs for stroke compared to naïve NSCs or vehicle control.

**Main body:**

Controlled studies investigating genetically modified NSC therapy using animal models of stroke were identified using PUBMED, Scopus and the Chinese National Knowledge Infrastructure (CNKI). Primary outcomes were lesion volume reduction and neurological functional recovery measured via Neurological Severity Score (NSS). Data for meta-analysis were synthesized and expressed as standardised mean difference (SMD) with 95% confidence intervals (CI) using inverse variance and a random effects model. Our secondary outcomes of interest included modified NSC survival, NSC differentiation into neurons, endogenous neural integration, anti-inflammatory effects and migratory capacities. Twenty-nine studies were included for systematic review, and eighteen studies were included for meta-analysis. Genetically modified NSCs significantly reduced lesion volume when compared to both naïve NSCs (SMD 1.07; 95% CI: [0.70, 1.43]; p < 0.00001) and vehicle/injury control (SMD 4.60; 95% CI: [2.69, 6.51]; p < 0.00001), and also significantly improved neurological functional recovery compared to both naïve NSCs (SMD 2.75; 95% CI: [0.34, 5.16]; p = 0.03) and vehicle/injury control (SMD 4.28; 95% CI: [0.64, 7.93]; p = 0.02).

**Conclusions:**

Existing preclinical literature suggests that genetically modified NSCs have the potential to improve therapeutic efficacy of NSCs. However, our systematic review revealed the field lacks uniformity in outcomes measured, an optimised protocol for cell therapy and absence of large animal model studies, all of which pose significant barriers to clinical translation and must be addressed for the field to progress. While we were unable to identify a clear candidate gene, our review highlights the potential for genetically modified NSCs as a future adjunct neuroprotective therapy for stroke and warrants further research in this area.

**Supplementary Information:**

The online version contains supplementary material available at 10.1186/s12967-025-07603-y.

## Introduction

Stroke is the most common cerebrovascular disease worldwide and is a leading cause of mortality and permanent neurological disabilities. In 2021 alone, there were 11.9 million new stroke cases and 7.3 million deaths from stroke worldwide [1]. The affected patient population is diverse, ranging from neonates to the elderly. Despite advances in gold standard therapies such as pharmacological thrombolysis and surgical mechanical thrombectomy, stroke patients that survive often experience high levels of neurological functional impairments [2]. Consequently, stroke contributes to 160.5 million disability adjusted life years (DALY).

The two major subtypes of stroke, ischaemic and haemorrhagic, cause a sudden decline in brain tissue perfusion, leading to a reduction in the supply of blood and oxygen to the brain, eventually causing neuronal cell death. Despite differing aetiologies, both subtypes share similar pathophysiological mechanisms that involve the process of neuroinflammation which exacerbates the primary injury, leading to an expansion of the irreversible injury core. Stroke pathophysiology consists of excitotoxicity, oxidative stress, inflammatory responses and blood brain barrier disruptions [3]. As part of the neuroinflammatory process, microglia, the primary immune cell of the brain, become activated and adopt a “M1-like” phenotype and increase the release of proinflammatory cytokines, resulting in recruitment of peripheral immune cells which infiltrate through the disrupted blood brain barrier [4, 5]. Altogether, ongoing neuroinflammation further propagates neuronal cell death and leads to secondary brain injury.

Existing gold standard therapies focus solely on restoring blood flow to salvage the penumbra surrounding the injury core. However, issues and complications such as reperfusion injury, haemorrhagic transformation of stroke and failed recanalization significantly limits treatment efficacy and recovery [6–9]. None of these therapies address the primary injury core. Moreover, emerging new neuroprotective agents have not yielded promising clinical trial results [10]. In recent decades, stem cell therapy has been viewed as a promising avenue as an adjunctive therapy with their regenerative and anti-inflammatory properties. Although there is a wide range of stem cell type and sources, neural stem cells (NSCs) exist endogenously in the human brain and have specific regenerative capacities for the central nervous system [11]. Endogenously, NSCs are found within the SVZ (subventricular zone) of the lateral ventricle and the SGZ (subgranular zone) of the dentate gyrus [12]. In response to specific environmental signals, NSCs can differentiate down either the neural lineage to become mature neurons, or the glial lineage to become astrocytes or oligodendrocytes [13]. However, in the event of a stroke, these endogenous NSCs alone are inadequate to mount a meaningful reparative response.

Early preclinical studies with NSCs excitingly demonstrated positive results in comparison to control using experimental stroke models, showing exogenous NSCs can replace lost cells and improve neurological functional outcomes [14]. Despite initial promise in preclinical settings, clinical trials have reported positive safety outcomes but mixed efficacy outcomes [15, 16]. Continued research in the field found graft NSCs survive poorly in the toxic ischaemic environment and have limited migratory abilities [17, 18]. These limitations create critical translational challenges. Thus, methods to overcome these limitations are being examined and one particular approach is through genetically engineering cells to enhance their effects. The present review aims to systematically evaluate the existing preclinical literature that compares the therapeutic effects of genetically modified NSCs as treatment for stroke to naïve NSCs or vehicle control, and to determine whether a promising candidate exists.

## Methods

A systematic review was conducted using the PRISMA methodology [19]. PICO for this study was specified as: Problem (P): animals of all ages with cerebral ischaemia; Intervention (I): NSCs genetically modified to overexpress various growth factors; Comparison (C): compared to control group with non-genetically modified neural stem cells or control virus modified neural stem cells or control group without treatment; and Outcome (O): reduction of lesion volume and/or functional recovery of studied animals.

### Search strategy

A systematic search was performed in PUBMED, Scopus and CNKI (China National Knowledge Infrastructure). Keywords related to cerebral ischemia and genetically modified neural stem cells were used to perform systematic searches within each database. The complete search strategy including Boolean strings can be found in Supplementary Data. To find additional articles, a manual search was conducted via Google Scholar, and reference lists of identified papers were also reviewed. The search period is all-time and not serialised. The most recent literature search using the above parameters was conducted March 2025.

### Selection criteria

Animal studies that investigated the use of genetically modified neural stem cells as therapy for cerebral ischemia were included in this review. Full-text in English and Chinese were included. Studies that only used wild type NSCs, only included in vitro work, did not have a control cell group, investigated modified microRNAs or review articles were excluded. Congress abstracts were excluded.

### Quality assessment and data extraction

All studies were imported into Covidence with duplicates removed before screening. Title and abstracts were screened by two independent researchers (S.L., RA.M.). Studies that met the inclusion criteria were included into full-text review. Conflicts were resolved by a third reviewer (C.M). Chinese full-texts were screened and extracted independently by two researchers who are native Chinese speakers (S.L., C.W.). All other data extraction were conducted independently by two researchers (S.L., C.M.). Extracted data from all included studies were author’s name, publication year, animal model characteristics, sample size, source of neural stem cell used, target gene modified, rationale for target gene selection, method of cell transfection, control group, timing of cell delivery, route of cell administration, total cell dose and use of immunosuppressants. Outcomes data extracted were in vitro tests, neural stem cell graft survival, graft migration, graft differentiation, lesion volume changes, graft impact on endogenous cells and behavioural tests of studied animals. Our Data extraction table template can be found in Supplementary Data.

The assessment of evidence quality was performed according to the Grading of Recommendations Assessment, Development and Evaluation (GRADE) process. GRADE score evaluation was conducted independently by two researchers (S.L., A.R.).

### Meta-analysis

A meta-analysis was conducted for the lesion volume and neurological functional recovery measured by a form of published neurological scoring system outcomes. A total of 14 studies were included for lesion volume, and 7 studies were included for neurological functional recovery measured. Where the study did not specify the type of data presented (mean or median and standard deviation or standard mean error), or did not specify the number of animals included, the study was excluded from the meta-analysis. Sub-group analysis was performed for total cell dose (≤1 M and > 1 M) and timing of cell administration (≤24 hours and > 24 hours).

### Risk of bias

Two authors (S.L., RA.M.) independently assessed risk of bias for all 29 studies included in this review using the Systematic Review Centre for Laboratory Animal Experimentation (SYRCLE) risk of bias tool [20], this tool has been validated and widely reviewed as the most appropriate risk of bias tool for animal studies. Biases assessed included: selection, performance, detection, attrition and reporting. Studies were graded with either: “Yes”, “No” or “Unclear”. Conflicts were resolved by a third author (C.M.).

### Statistical analysis

Where raw values were not available, two researchers independently extracted numerical data from graphs using Plot Digitizer (S.L., C.M.) and values were averaged. Discrepancies of over 1 point were resolved through a third researcher (RA.M.) conducting an independent extraction. Data were recorded as mean and standard deviation of lesion volume and functional recovery score. Cochrane Review Manager (revMan) version 7.12.0 was used to generate forest plots. Standard mean difference with 95% confidence interval (95% CI) was reported using inverse variance. Due to the vast heterogeneity between studies observed during data extraction, a random-effect model was used. Heterogeneity among studies was estimated using the Restricted Maximum-Likelihood method. GraphPad Prism version 10.4.2 was used to generate other graphs.

## Results

### Study selection

**Fig. 1:**
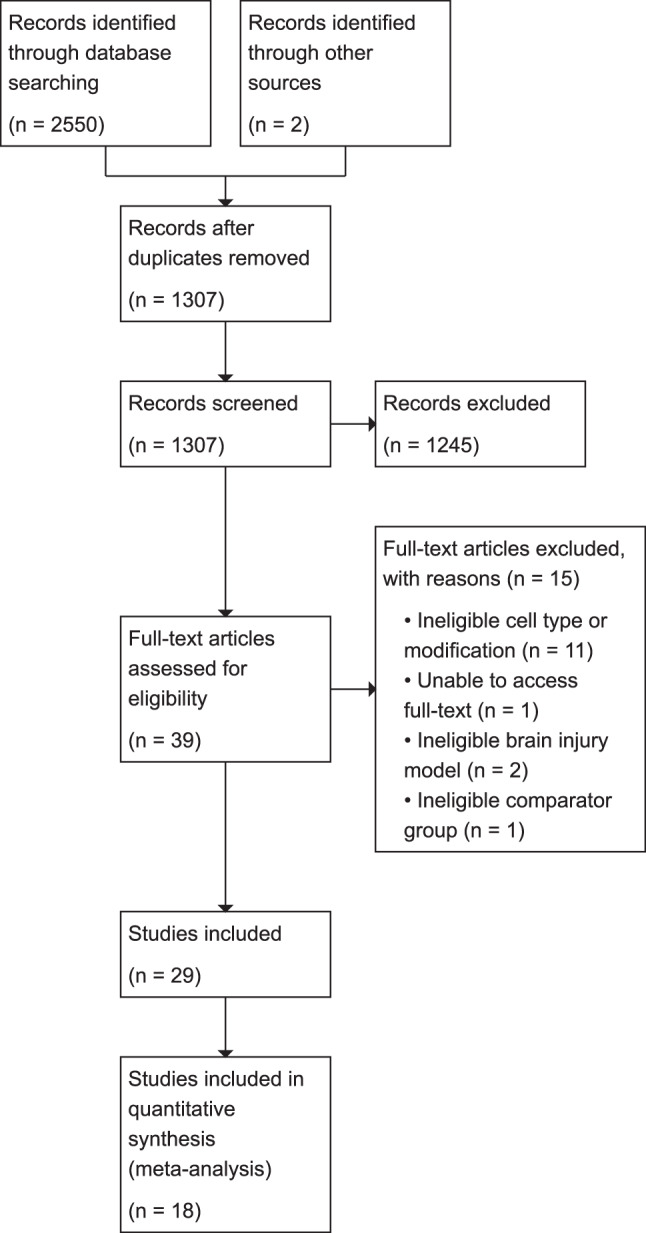
PRISMA chart

A total of 2547 records were identified following the search method shown in Fig. 1. After duplicates were removed, 1307 studies were screened by title and abstract. 1245 studies were excluded. Full-text screening was performed on 39 studies, and 29 studies met the inclusion criteria and were included in this systematic review. 18 studies were included in the meta-analysis. Updated searches yielded 2 additional records which met our inclusion criteria and were all included.

### Study characteristics

A total of 29 studies were included in this review, see Table 1 for study characteristics. All included studies were performed on rodent injury models (16 rats and 13 mice). No large animal model studies were found. Animal models used included MCAO - temporary or permanent (middle cerebral artery occlusion, *n* = 22), ICH (intracerebral haemorrhage, *n* = 4), HIE (Hypoxic-ischaemic encephalopathy, *n* = 3). Most studies investigated adult rodent models; four studies investigated neonatal rodents using HIE models. Most studies used NSCs sourced from rodents (*n* = 25). Only four studies used human sourced NSCs, and three of these studies were conducted by the same laboratory group and used the same foetal human NSC cell line. The other study used both a commercial human cell line and NSC derived from human ESC (embryonic stem cell). Most studies utilised some form of viral gene transfection (*n* = 24). The remaining studies used either genetically modified animals to source NSCs (*n* = 3); plasmid and scaffold (*n* = 1); or genetically modified embryonic stem cells (*n* = 1).

**Table 1:** Included study characteristics

Author, Year	Injury Model	Animal Age	Species	Cell Source	Target Gene	Method of modification		Comparator	Cell Administration time post-injury	Route of Administration	Immunosuppression	Total Cell Dose
Andsberg G et al. 1998 [21]	tMCAO (temporary)	Adult	Rat	Rat	NGF		Retrovirus	Injury + Naïve NSCs, Injury + saline	7 days prior	Intracerebral	No	9 ×10^5^
Bernstock JD et al. 2019 [22]	tMCAO (temporary)	Adult	Mouse	Mouse	Ubc9		Modified mice	Injury + Naïve NSC	3 days	Intracerebral	No	1 ×10^5^
Chen B et al. 2009 [23]	tMCAO (temporary)	Adult	Rat	Rat	GDNF		Adenovirus	Injury + Naïve NSCs, Injury + saline	3 days	Intraventricular	No	5 ×10^5^
Dayer AG et al. 2007 [24]	HIE	PND3	Rat	Rat	bFGF		Lentivirus	Injury + Naïve NSC	Postnatal day 3 (D0 of injury)	Intracerebral	No	N/A
Jenny B et al. 2009 [25]	HIE	PND3	Rat	Rat	bFGF		Lentivirus	Injury + Naïve NSC	4 days prior	Intracerebral	No	2 - 5 × 10^4^
Jiang XC et al. 2019 [26]	MCAO	N/A	Mouse	Mouse	BDNF		Plasmid	Injury + Naïve NSC, Injury + no vehicle	1 day	Intravenous	No	3 - 5 × 10^5^
Kameda M et al. 2007 [27]	MCAO	Adult	Rat	Rat	GDNF		Adenovirus	Injury + Naïve NSCs	3 hours	Intracerebral	No	3 ×10^5^
Korshunova I et al. 2020 [28]	MCAO (distal)	Adult	Mouse	Human	BCL2L1		Lentivirus	Injury + Naïve NSCs, Injury + saline	2 days	Intracerebral	Yes	5 ×10^4^
Lee HJ et al. 2007 [29]	ICH	Adult	Mouse	Human	VEGF		Retrovirus	Injury + Naïve NSCs, Injury + saline	7 days	Intracerebral	No	2 ×10^5^
Lee HJ et al. 2009 [30]	ICH	N/A	Mouse	Human	Akt1		Retrovirus	Injury + Naïve NSCs, Injury + saline	7 days	Intracerebral	No	2 ×10^5^
Lee HJ et al. 2010 [31]	ICH	N/A	Mouse	Human	BDNF		Retrovirus	Injury + Naïve NSCs, Injury + saline	7 days	Intracerebral	No	2 ×10^5^
Liu F et al. 2020 [32]	MCAO	Adult	Rat	Rat	GDNF		Adenovirus	Injury + Naïve NSCs, Injury + saline	3 days	Intracerebral	No	5 ×10^5^
Pignataro G et al. 2007 [33]	tMCAO (temporary)	N/A	Mouse	Mouse	Adenosine release		Plasmid	Injury + Naïve NSCs, Injury + saline	7 days prior	Intracerebral	No	5 ×10^4^
Sakata H et al. 2012 [34]	tMCAO (temporary)	Adult	Mouse	Mouse	SOD1		Modified mice	Injury + Naïve NSCs	2 days	Intracerebral	No	3 ×10^5^
Song W et al. 2024 [35]	MCAO	Adult	Rat	Mouse	BDNF		Lentivirus	Injury + Naïve NSC, Injury + no vehicle	2 hours	Intracerebral	No	5 ×10^5^
Tang X et al. 2023 [36]	MCAO (Brainstem)	Adult	Mouse	Mouse	BDNF		Lentivirus	Injury + Naïve NSC	7 days	Intracerebral	No	1 ×10^6^
Tian L et al. 2019 [37]	tMCAO (temporary)	Adult	Rat	Mouse	LIF		Lentivirus	Injury + Naïve NSCs, Injury + saline	6 hours	Intravenous	No	5 ×10^6^
Wakai T et al. 2013 [38]	ICH	Adult	Mouse	Mouse	Sod1		Modified mice	Injury + Naïve NSCs, Injury + saline	3 days	Intracerebral	No	2 ×10^5^
Wang J et al. 2015 [39]	tMCAO (temporary)	N/A	Mouse	Mouse	Gal-1		Adenovirus	Injury + saline	2 hours	Intracerebral	No	1 ×10^5^
Wu W et al. 2010 [40]	tMCAO (temporary)	Adult	Rat	Rat	HIF-1a		Adenovirus	Injury + Naïve NSCs, Injury + saline	1 day	Intracerebral	No	1 ×10^6^
Wu Z 2015 [41]	MCAO	Adult	Rat	Rat	GDNF		Adenovirus	Injury + Naïve NSCs, Injury + saline	3 days	Intraventricular	No	5 ×10^5^
Xu P et al. 2019 [42]	tMCAO (temporary)	Adult	Mouse	Mouse	BRCA1		Lentivirus	Injury + Naïve NSCs, Injury + saline	6 hours	Intracerebral	No	6 ×10^5^
Yao Y et al. 2016 [43]	HIE	PND7	Rat	Rat	VEGF		Lentivirus	Injury + Naïve NSCs, Injury + saline	3 days	Intracerebral	No	1 ×10^5^
Ye Q et al. 2017 [44]	MCAO (permanent)	PND9	Mouse	Mouse	bFGF		Adenovirus	Injury + Naïve NSCs, Injury + saline	3 days	Intranasal	No	1 ×10^6^
Zhang JJ et al. 2017 [45]	tMCAO (temporary)	Adult	Rat	Mouse	bFGF		Lentivirus	Injury + Naïve NSCs, Injury + saline	1 day	Intravenous	No	5 ×10^6^
Zhang Z et al. 2008 [46]	MCAO	Adult	Rat	Rat	NT-3		Lentivirus	Injury + Naïve NSCs, Injury + saline	7 days	Intracerebral	No	2 ×10^5^
Zhu J et al. 2017 [47]	tMCAO	Adult	Rat	Rat	Noggin		Adenovirus	Injury + Naïve NSC, Injury + no vehicle	3 days	Intraventricular	No	5 ×10^5^
Zhu JM et al. 2011 [48]	tMCAO (temporary)	Adult	Rat	Rat	BDNF		Retrovirus	Injury + Naïve NSCs, Injury + saline	3 days	Intracerebral	No	1 ×10^6^
Zhu Y et al. 2024 [49]	tMCAO	Adult	Rat	Rat	VEGF		Adenovirus	Injury + Naïve NSC, Injury + no vehicle	3 days	Intracerebral	No	5 ×10^6^

Abbreviations: Akt1, RAC (Rho family)-alpha serine/threonine-protein kinase; BCL2L1, Bcl-2-like protein 1; BDNF, brain-derived neurotrophic factor; bFGF, basic fibroblast growth factor; BRCA1, breast cancer gene 1; Gal-1, galectin 1; GDNF, glial cell line-derived neurotrophic factor; HIF-1a, hypoxia-inducible factor 1-alpha; LIF, leukemia inhibitory factor; NT-3, neurotrophin-3; NGF, nerve growth factor; SOD1, superoxide dismutase 1; Ubc9, SUMO-conjugating enzyme ubiquitin carrier protein 9; VEGF, vascular endothelial growth factor. N/A used where study did not specify age of animals

The target gene selected greatly differed across all studies. The most common target genes were BDNF (*n* = 5), GDNF (*n* = 4), bFGF (*n* = 4) and VEGF (*n* = 3). Despite heterogeneity among the selected genes of interest, there were consistent themes behind the authors’ rationale. Most were aimed at improving cell survival in a toxic ischaemic environment, mostly via modulation of neuroinflammation (*n* = 24). In comparison, a few studies targeted enhanced neurogenesis (*n* = 3). While studies also targeted enhanced neuronal differentiation (*n* = 3), one study aimed at improving the retainment of the immature stem cell nature of NSCs.

#### Intervention methods

**Fig. 2:**
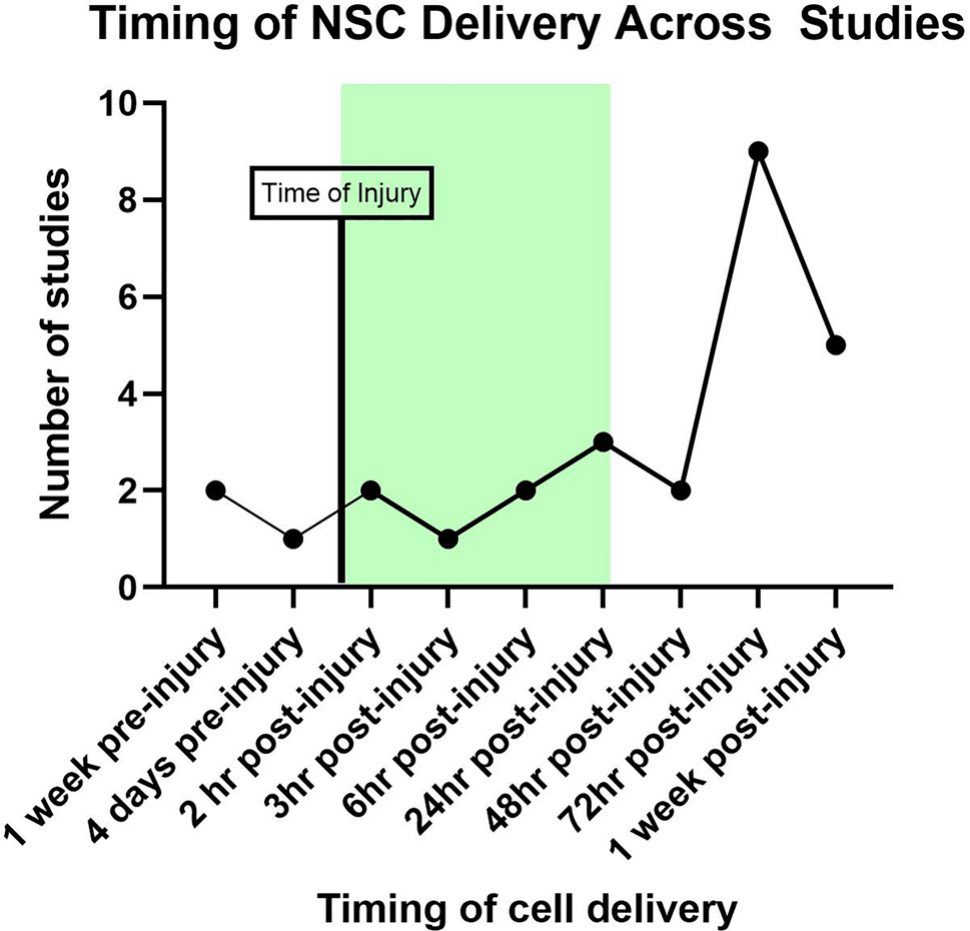
Timing of NSC delivery across included studies. Green area highlights the acute treatment phase which matches with current clinical treatment guidelines

Intracerebral was the most common route of cell administration (*n* = 22), followed by both intraventricular (*n* = 3) and intravenous (*n* = 3), with intranasal least common (*n* = 1), no studies used intra-arterial delivery. Timing of cell therapy delivery varied greatly across all studies (Fig. 2). Three studies treated animals with NSCs prior to injury, five studies targeted the acute injury period within the first 6 hours of injury, three studies treated animals between 6 hours and 24 hours, 12 studies treated animals between 24 hours to 72 hours, and 5 studies delivered treatment 4 days and later, while one study did not report timing of NSC delivery. The use of immunosuppressants, a commonly used co-therapy with naïve NSCs to improve cell engraftment and retention, was only reported in one study. Dosages also varied greatly across studies, ranging from 5 × 10^4^ cells to 5 × 10^6^ cells and one neonatal HIE model study cell dosage was not reported.

### Analysis of primary outcomes - lesion volume and functional recovery

The results of the primary outcomes for this systematic review are summarised in Table 2.

**Table 2:** Summary of primary outcomes of included studies

Author, Year	Genetic modification	Lesion Volume Reduction Latest Timepoint	Method of Measurement	Neurological Scoring System	Functional Outcome
Andsberg G et al. 1998 [21]	NGF	−	DARPP-32 & NeuN	−	De Ryck et al. score NS: 24 hours
Bernstock JD et al. 2019 [22]	Ubc9	NS: 30 days	Cresyl violet stain	−	−
Chen B et al. 2009 [23]	GDNF	Significant: 1 week	H & E stain	mNSS Scale (0–18)	Significant: 2–3 weeks
Dayer AG et al. 2007 [24]	bFGF	−	−	−	−
Jenny B et al. 2009 [25]	bFGF	−	−	−	−
Jiang XC et al. 2019 [26]	BDNF	Reduced: 4 weeks	TTC stain	mNSS Scale (0–18)	Significant: 4 weeks
Kameda M et al. 2007 [27]	GDNF	Significant: 1 week	MRI	−	Limb placement & Cylinder. Significant: 1 week
Korshunova I et al. 2020 [28]	Bcl2l1	−	−	−	Latency to move, Grabbing attempts, Collins score. Significant: 1 week
Lee HJ et al. 2007 [29]	VEGF	−	−	−	Rotarod, Limb placement. Significant: 3–9 weeks
Lee HJ et al. 2009 [30]	Akt1	−	−	−	Rotarod, Limb placement. Significant: 5 weeks (rotarod), 2–8 weeks (Limb placement)
Lee HJ et al. 2010 [31]	BDNF	−	−	−	Rotarod, Limb placement. Significant: 3–9 weeks
Liu F et al. 2020 [32]	GDNF	−	Nerve fibre damage	−	−
Pignataro G et al. 2007 [33]	Adenosine release	Significant: 1 week	TTC stain	General score (0–28) and focal neurologic score (0–28)	Focal score significant: 24 hours
Sakata H et al. 2012 [34]	SOD1	Significant: 8 weeks	H & E stain	mNSS Scale (0–14)	NS: 4 weeks
Song W et al. 2024 [35]	BDNF	Significant: 5 days	TTC stain	Zea-Longa scale (0–5)	Significant: 5 days
Tang X et al. 2023 [36]	BDNF	Significant: 4 weeks	Number of neurons	Voetsch score (0–50)	Improved: 4 weeks
Tian L et al. 2019 [37]	LIF	Significant: 4 weeks	MRI	mNSS scale (0–15)	Significant: 2 weeks
Wakai T et al. 2013 [38]	SOD1	Significant: 5 weeks	Cresyl violet stain	−	Cylinder test Significant: 5 weeks Corner turn test: NS
Wang J et al. 2015 [39]	Gal-1	Significant: 4 weeks	MAP2 area	−	Cylinder test Significant: 3 days Morris water maze: NS 4 weeks
Wu W et al. 2010 [40]	HIF-1a	−	−	mNSS scale (0–18)	Significant: 28 days
Wu Z 2015 [41]	GDNF	Significant: 1 week	H & E stain	mNSS scale (0–18)	Significant: 3 weeks
Xu P et al. 2019 [42]	BRCA1	−	−	mNSS scale (0–10)	Significant: 4 weeks
Yao Y et al. 2016 [43]	VEGF	−	Number of neurons	−	Radial arm maze, Attitudinal reflex NS: 30 days
Ye Q et al. 2017 [44]	bFGF	Significant: 5 weeks	MAP2 & MBP stain	−	Cylinder test. Significant: 5 weeks Adhesive removal. NS: 5 weeks
Zhang JJ et al. 2017 [45]	bFGF	NS: 1 week	TTC stain	NSS scale (0–15)	Significant: 4 weeks
Zhang Z et al. 2008 [46]	NT-3	−	−	NSS scale (0–15)	Significant: 2 weeks
Zhu J et al. 2017 [47]	Noggin	Significant: 24 hours	TTC stain	Zea Longa scale (0–5)	Significant: 4 weeks
Zhu JM et al. 2011 [48]	BDNF	−	−	NSS Scale (0–18)	Significant: 12 weeks
Zhu Y et al.[49]	VEGF	Significant: 1 week	TTC stain	Zea Longa scale (0–5)	Significant: 1 week

Note: (-) indicates study did not measure this outcome, NS = not statistically significant, Significant = statistically significant compared to naïve NSCs

Abbreviations: DARPP-32, dopamine and cAMP-regulated phosphoprotein; H&E, hematoxylin and eosin; MAP2, microtubule-associated protein 2; MBP, myelin basic protein; mNSS, modified neurological severity score; MRI, magnetic resonance imaging; NeuN, neuronal nuclei; NSS, neurological severity score; TTC, 2,3,5-Triphenyltetrazolium chloride

Abbreviations for genetic modifications: Akt1, RAC (Rho family)-alpha serine/threonine-protein kinase; BCL2L1, Bcl-2-like protein 1; BDNF, brain-derived neurotrophic factor; bFGF, basic fibroblast growth factor; BRCA1, breast cancer gene 1; Gal-1, galectin 1; GDNF, glial cell line-derived neurotrophic factor; HIF-1a, hypoxia-inducible factor 1-alpha; LIF, leukemia inhibitory factor; NT-3, neurotrophin-3; NGF, nerve growth factor; SOD1, superoxide dismutase 1; Ubc9, SUMO-conjugating enzyme ubiquitin carrier protein 9; VEGF, vascular endothelial growth factor

**Fig. 3:**
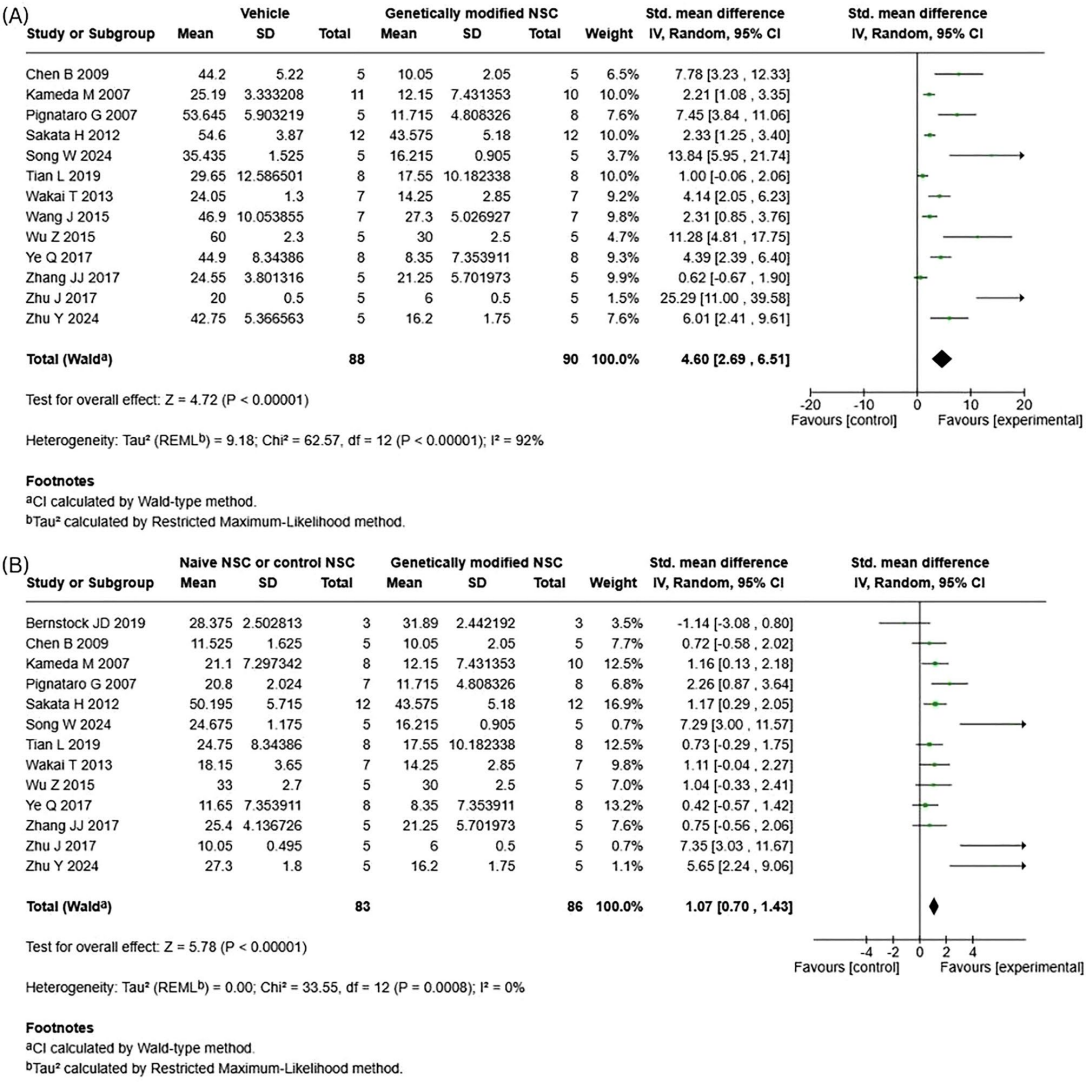
Forest plot for lesion volume reduction. **A**) Vehicle/Injury control vs genetically modified NSCs. **B**) naive NSCs vs genetically modified NSCs

A total of 18 studies measured lesion volume reduction. 14 studies met inclusion criteria for our meta-analysis and measured lesion volume reduction using either histology (*n* = 12) or MRI (*n* = 2). Our meta-analysis found that genetically modified NSCs result in statistically significant improvements in lesion volume when compared to both vehicle/injury control and naïve NSCs. The greatest benefits were observed when genetically modified NSC treatment was compared to vehicle/injury control, (Fig. 3A; Std mean difference (SMD) 4.60; 95% CI: [2.69, 6.51]; *p* < 0.0001). When genetically modified NSCs were directly compared to naïve NSC treatment, genetically modified NSC treatment had a statistically significant reduction in lesion volume (Fig. 3B; SMD 1.07; 95% CI: [0.70, 1.43]; *p* < 0.00001). Significant heterogeneity was observed for both meta-analyses, the heterogeneity score was high when genetically modified NSCs were compared to vehicle (Fig. 3A; I^2^ = 92%) and low when compared to naïve NSCs (Fig. 3B; I^2^ = 0%). The funnel plot for vehicle versus genetically modified NSCs showed pronounced right-sided asymmetry (Supp. Fig. 1A), with small studies reporting disproportionately large effects and an extreme outlier, indicating strong evidence of publication bias or small-study effects. In contrast, the funnel plot for naïve versus genetically modified NSCs (Supp. Fig. 1B) was largely symmetrical with only mild imbalance, suggesting minimal evidence of publication bias in this comparison.

A total of 16 studies utilised a published neurological functional recovery scoring system to investigate the effect of genetically modified NSCs on neurological functions. Of these, 7 studies met our inclusion criteria for meta-analysis, though these studies reported slightly differing scoring systems; modified neurological severity score (mNSS) 18 points (*n* = 3), mNSS 15 points (*n* = 2), mNSS 10 points (*n* = 1) and Zea Longa Score 5 points (*n* = 1).

**Fig. 4:**
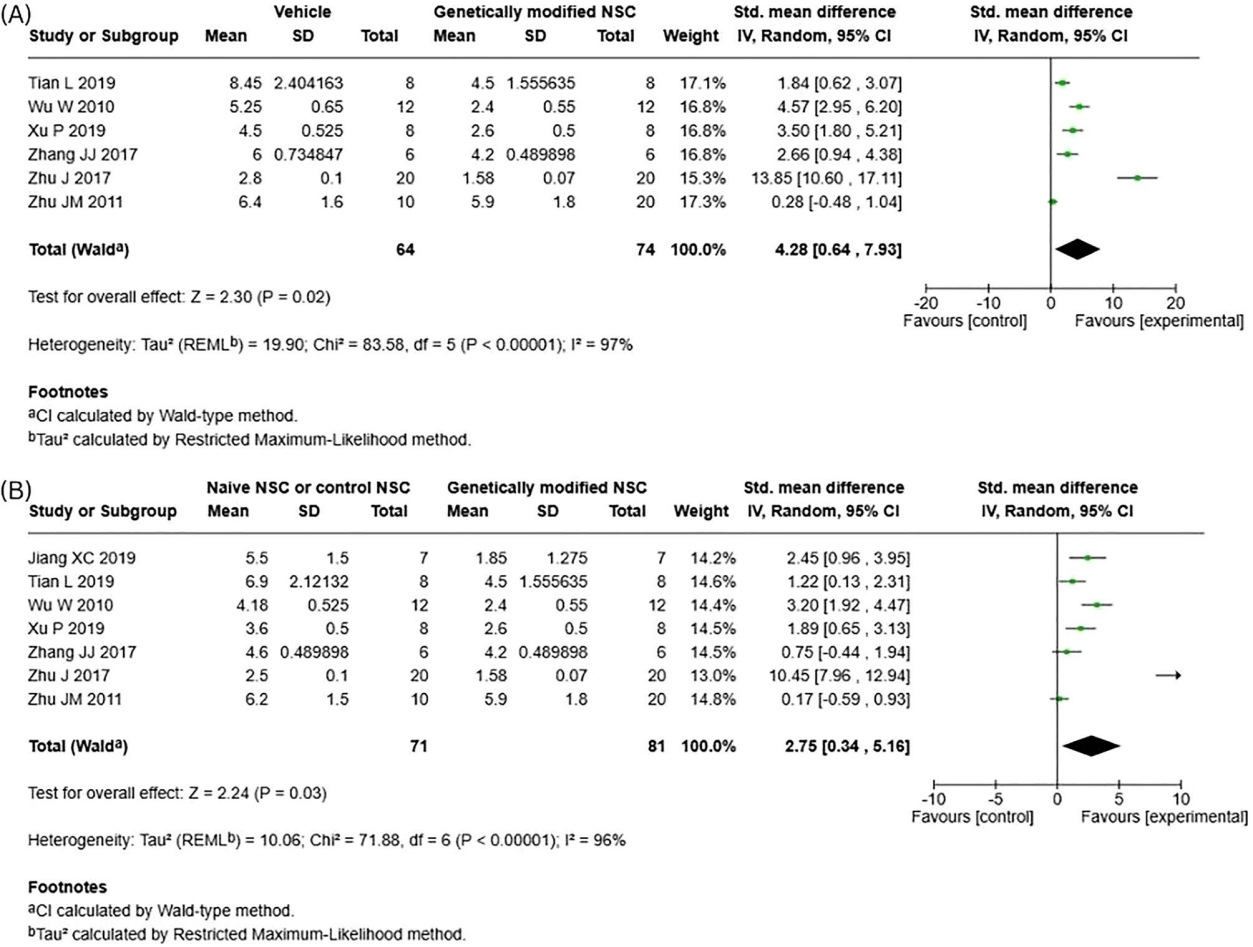
Forest plot for neurological functional recovery. **A**) Vehicle/Injury control vs genetically modified NSCs. **B**) naive NSCs vs genetically modified NSCs

For functional recovery, our meta-analysis revealed that genetically modified NSCs led to a significant improvement in neurological function compared to vehicle/injury control (Fig. 4A; SMD 4.28; 95% CI: [0.64, 7.93]; *p* = 0.02). Importantly, genetically modified NSCs also significantly improved functional recovery compared to naïve NSCs (Fig. 4B; SMD 2.75; 95% CI: [0.34, 5.16]; *p* = 0.03). Significant heterogeneity was observed in both meta-analysis and I^2^ scores were high for both (I^2^ = 97 and 96%, respectively). As fewer than 10 studies were eligible, publication bias could not be assessed with funnel plots for neurological functional recovery or its subgroups.

#### Subgroup analysis – cell dose

**Fig. 5:**
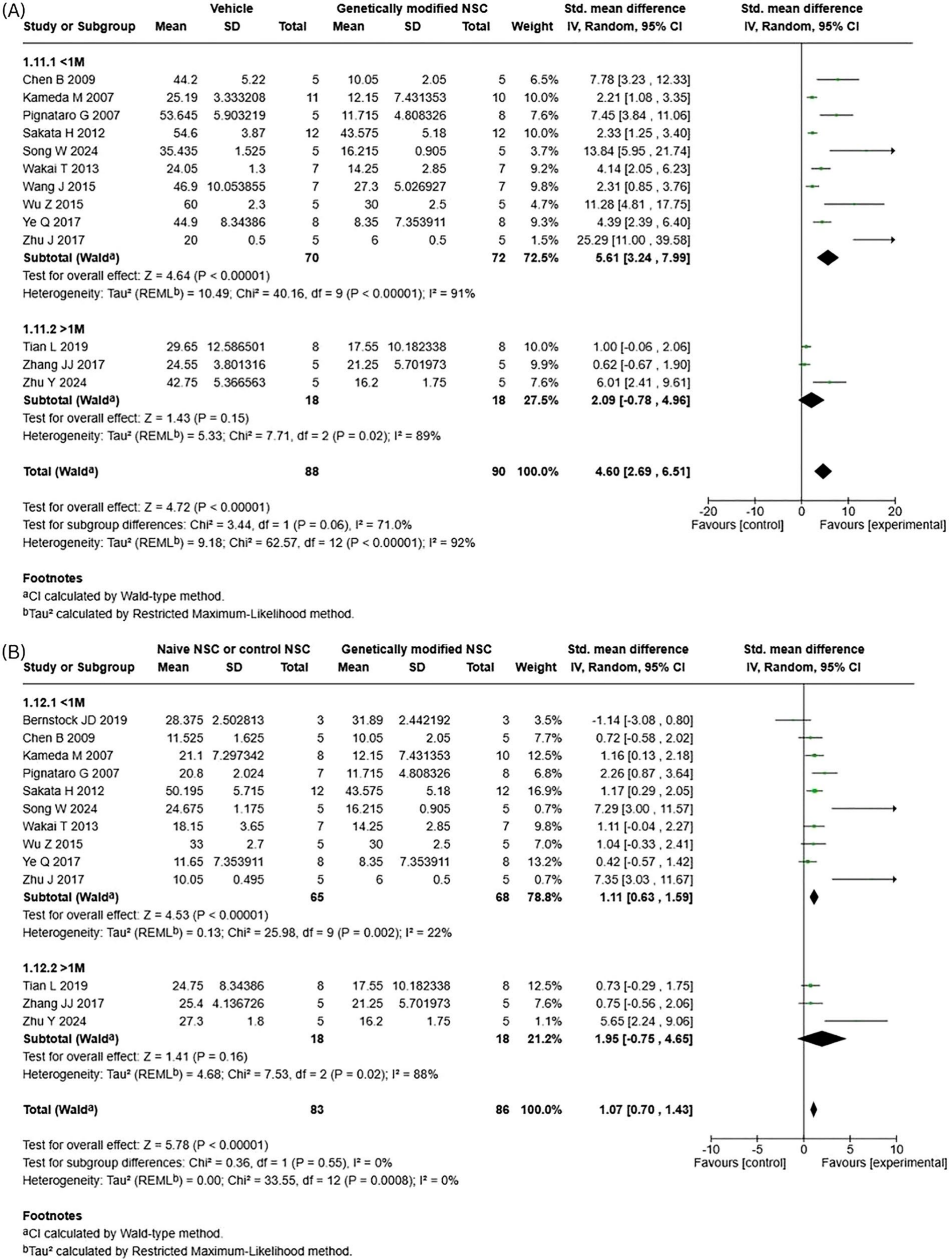
Subgroup analysis for lesion volume reduction – cell dose. (**A**) vehicle vs genetically modified NSCs. (**B**) Naïve NSCs vs genetically modified NSCs

To examine if cell dose plays a role in the therapeutic efficacy of genetically modified NSCs in stroke, studies included for each meta-analysis were grouped by cell dose of less than or equal to 1x10^6^ and above 1x10^6^ total cells. For lesion volume reduction (Fig. 5), subgroup analysis revealed no significant subgroup differences for genetically modified NSCs compared to vehicle (Fig. 5A; *p* = 0.06, I^2^ = 71%) and naïve NSCs (Fig. 5B; *p* = 0.55, I^2^ = 0%). However, when genetically modified NSCs were compared to both vehicle (Fig. 5A; SMD 2.09; 95% CI: [−0.78, 4.96]; *p* = 0.15, I^2^ = 89%) and naïve NSCs (Fig. 5B; SMD 1.95; 95% CI: [−0.75, 4.65]; *p* = 0.16, I^2^ = 88%), doses of over 1 M cells did not have a statistically significant effect. Whereas a statistically significant effect was observed when a total cell dose of 1 M or less was compared to both vehicle (Fig. 5A; SMD 5.61; 95% CI: [3.24, 7.99]; *p* < 0.00001, I^2^ = 91%) and naïve NSCs (Fig. 5B; SMD 1.11; 95%CI: [0.63, 1.59]; *p* < 0.00001, I^2^ = 22%). For the vehicle versus genetically modified NSCs, the funnel plot showed marked right-sided asymmetry across both < 1 M and > 1 M cell-dose subgroups (Supp. Fig. 2A), with small studies clustering around large positive effects and a notably extreme outlier, indicating substantial publication bias or small-study effects. In contrast, the naïve versus genetically modified NSC comparison displayed a more symmetrical distribution across dose subgroups (Supp. Fig. 2B), with only minor imbalance and no extreme outliers, suggesting minimal evidence of publication bias in this analysis.

**Fig. 6:**
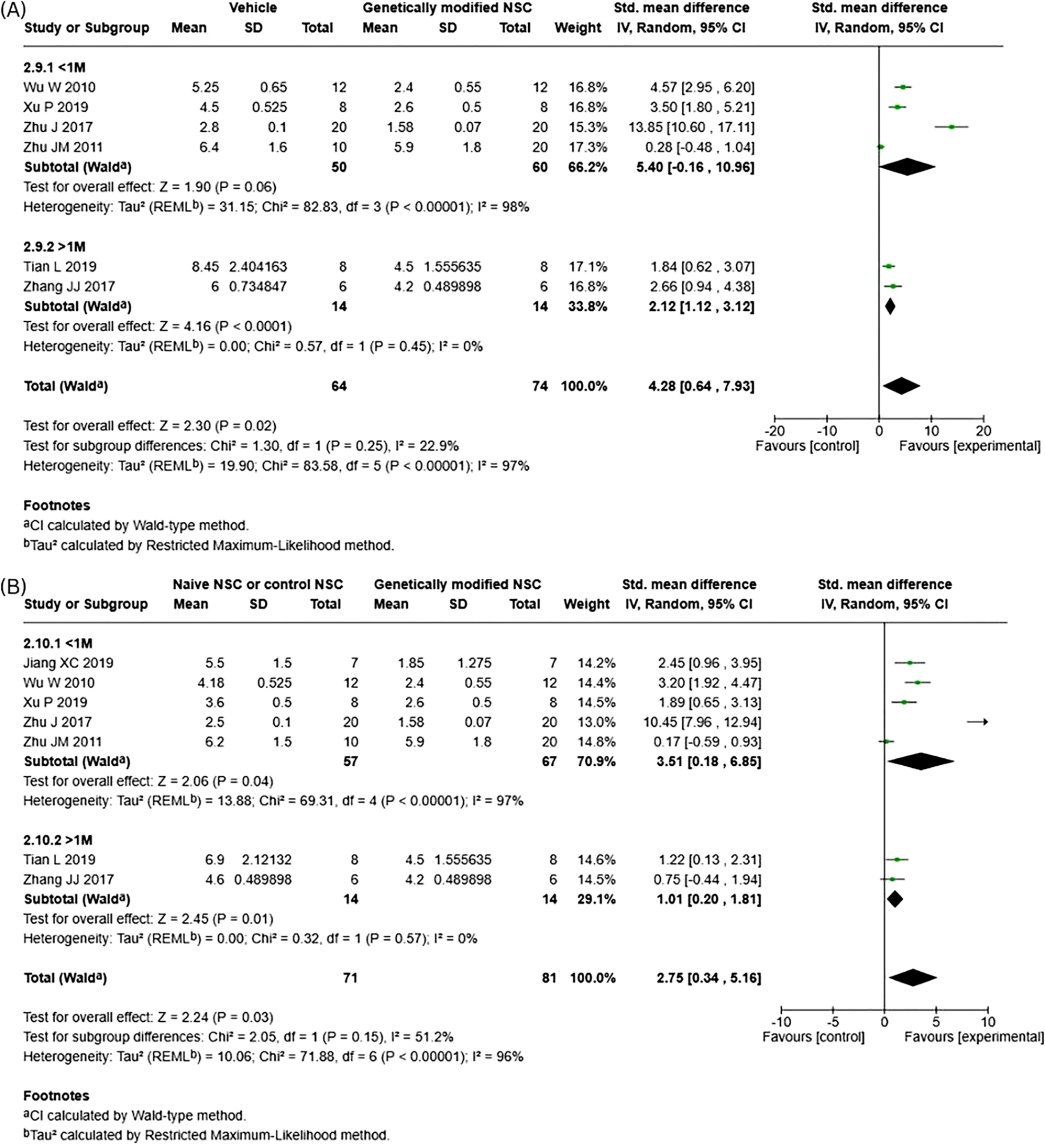
Subgroup analysis for neurological functional recovery – cell dose. (**A**) vehicle vs genetically modified NSCs. (**B**) Naïve NSCs vs genetically modified NSCs

Subgroup analysis showed no significant subgroup differences between the cell doses for neurological functional recovery when comparing genetically modified NSCs to both vehicle (Fig. 6A; *p* = 0.25, I^2^ = 22.9%) and naïve NSCs (Fig. 6B; *p* = 0.15, I^2^ = 51.2%). When genetically modified NSCs were compared to vehicle, total cell dose of 1 M or less did not result in statistically significant differences (SMD 5.40; 95% CI: [−0.16, 10.96]; *p* = 0.06, I^2^ = 98%). However, when compared to naïve NSCs, a statistically significant difference was seen (SMD 3.51; 95% CI: [0.18, 6.85]; *p* = 0.04, I^2^ = 97%). For total cell dose of over 1 M, statistically significant differences were seen when compared to both vehicle (SMD 2.12; 95% CI: [1.12, 3.12]; *p* < 0.0001, I^2^ = 0%) and naïve NSCs (SMD 1.01; 95% CI: [0.20, 1.81]; *p* = 0.01, I^2^ = 0%). It is important to note, that across all subgroup comparisons, heterogeneity remained high.

#### Subgroup analysis – timing of administration

**Fig. 7:**
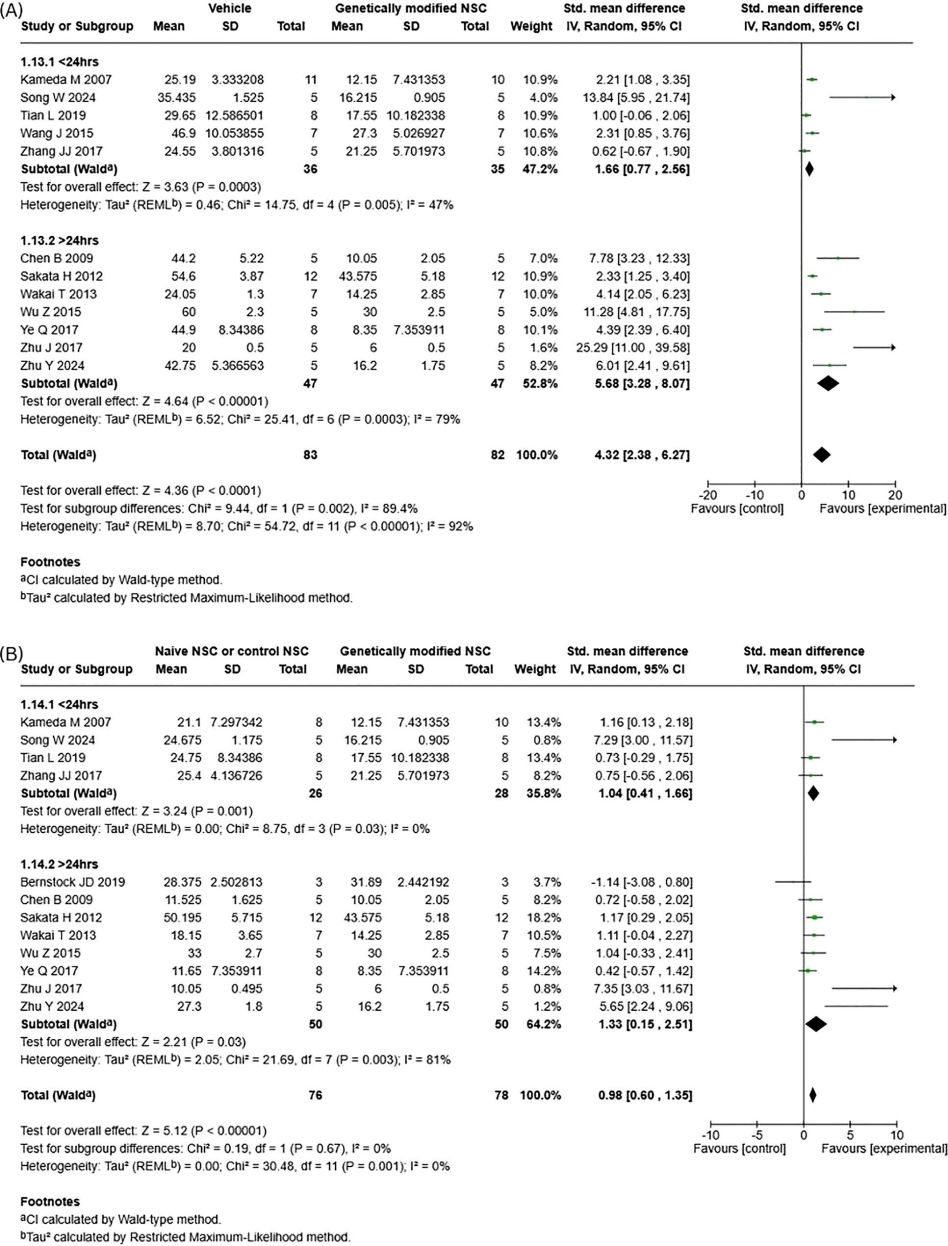
Subgroup analysis for lesion volume reduction – timing of NSC administration. (**A**) vehicle vs genetically modified NSCs. (**B**) Naïve NSCs vs genetically modified NSCs

To examine if timing of treatment plays a role in the therapeutic efficacy of genetically modified NSCs in stroke, studies included for analysis were grouped by timing of cell administration post-injury; 24 hours or less (*n* = 5) and over 24 hours (*n* = 8). Subgroup difference analysis of timing of cell administration showed differing results between lesion volume reduction and neurological function recovery. For lesion volume, there was a significant subgroup difference between cell administration given before 24 hours compared to after 24 hours for genetically modified NSCs compared to vehicle (Fig. 7A, *p* = 0.002, I^2^ = 89.4%), with treatment post 24 hours leading to better outcomes. This same effect was not seen when genetically modified NSCs were compared to naïve NSCs (Fig. 7B, *p* = 0.67, I^2^ = 0%). When assessing all subgroups independently for reduction of lesion volume, all groups, regardless of timing of administration, were found to have a statistically significant difference (Fig. 7A, B). The vehicle versus genetically modified NSC funnel plot showed clear right-sided asymmetry across both timing subgroups (Supp. Fig. 3A), including an extreme outlier, indicating strong publication bias or small-study effects. In contrast, the naïve versus genetically modified NSC plot was more symmetrical with only mild imbalance (Supp. Fig. 3B), suggesting minimal publication bias.

**Fig. 8:**
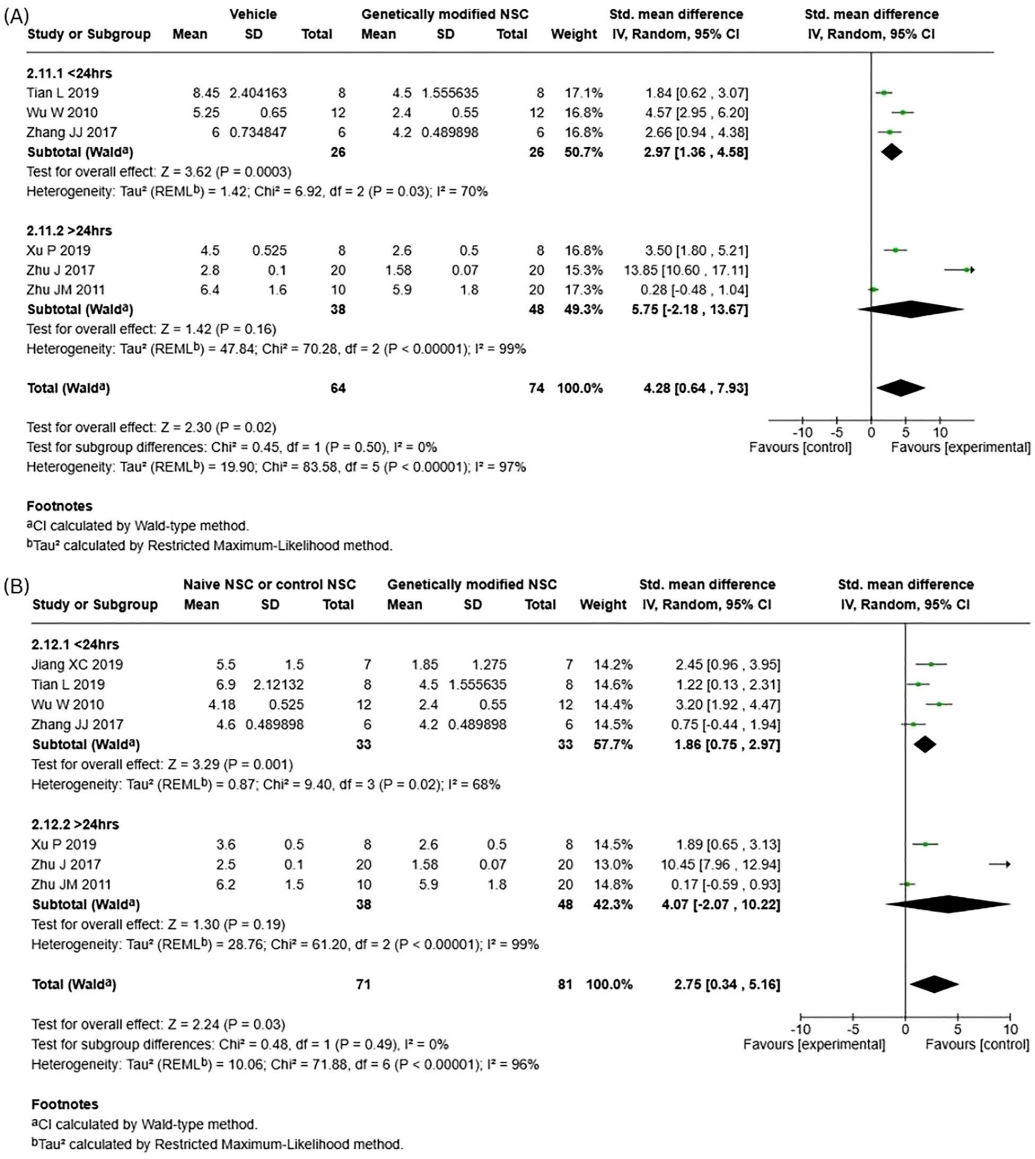
Subgroup analysis for neurological functional recovery – timing of NSC administration. (**A**) vehicle vs genetically modified NSCs. (**B**) Naïve NSCs vs genetically modified NSCs

In comparison, no significant subgroup differences were observed for neurological functional recovery for genetically modified NSCs compared to vehicle (Fig. 8A, *p* = 0.5, I^2^ = 0%), or naïve NSCs (Fig. 8B, *p* = 0.49, I^2^ = 0%). Assessment of individual subgroup analysis revealed that treatment after 24 hours did not significantly improve neurological function when genetically modified NSCs were compared to vehicle (SMD 5.75; 95% CI: [−2.18, 13.67]; *p* = 0.16, I^2^ = 99%), or naïve NSCs (SMD 4.07; 95% CI: [−2.07, 10.22]; *p* = 0.19, I^2^ = 99%). While treatment before 24 hours resulted in a statistically significant mean difference when genetically modified NSCs were compared to vehicle (SMD 2.97; 95% CI: [1.36, 4.58]; *p* = 0.0003, I^2^ = 70%) and naïve NSCs (SMD 1.86; 95% CI: [0.75, 2.97]; *p* = 0.001, I^2^ = 68%).

To further explore factors influencing lesion volume and neurological functional recovery, we conducted subgroup analyses examining the injury model (Supp. Fig. 4–6, route of administration (Supp. Fig. 7–9), and specific genetic variations (e.g., BDNF, bFGF; Supp. Fig. 10–13). No significant differences were found across subgroups, except for genetic variation; however, most genetic variation subgroups included only a single study, limiting the strength of the evidence, and preventing definitive conclusions.

## Secondary outcomes of interest

### Graft survival

A key goal of genetically modifying NSCs is to improve their survival in the ischaemic stroke environment to improve the therapeutic potential of the cells. Our systematic review sought to compare the outcomes of genetically modified NSCs on graft survival when assessed at an early time-frame (within 28 days) and late time-frame (28 days and over) post cell administration (Table 3). However, due to high heterogeneity between studies that measured cell survival, a meta-analysis could not be performed. A total of 17 studies measured differences in graft NSC survival between genetically modified and naïve NSCs. Of these, 11 studies reported outcomes for early time-frame survival. Majority of the studies reported genetically modified NSCs to have significantly higher graft survival than naïve NSCs. Dayer AG et al. (bFGF) found the genetically modified NSCs with bFGF led to a 2.4-fold increase in cell survival compared to the naïve NSC group [24]. The group of Lee HJ et al. conducted three different studies investigating three different genetic modifications [29–31]. All three studies found the genetically modified NSCs led to improved survival; 68% vs. 35.6% (VEGF) [29], 54% vs. 39% (Akt1) [30] and 52.7% vs. 32% (BDNF) [31]. Similarly NSC survival was shown to significantly increase with modifications including Ubc9 [22], Bcl2l1 [28], GDNF [23, 41], Sod1 [38] and BDNF [26]. Only one study reported no significant differences in cell survival between genetically modified NSCs with GDNF and naïve NSCs [27]. Overall, these findings suggest that genetically modified NSCs may have a better chance of surviving the toxic environment compared to naïve NSCs.

**Table 3:** Summary of secondary outcomes of included studies

Author, Year	Genetic modification	Grafted modified NSC Survival - last timepoint detected	Graft modified NSC Differentiation	Endogenous Neural Integration	Anti-inflammatory Effects	Modified Cell Migration
Andsberg G et al. 1998 [21]	NGF	48 hours	−	−	−	1–1.5 mm from injection site
Bernstock JD et al. 2019 [22]	Ubc9	−	✓ MAP2	✓ PSD-95	✓ Reduced Caspase-3	−
Chen B et al. 2009 [23]	GDNF	5 days	−	✓ PSD-95 ✓ Syp protein	✓ Reduced Caspase-3 ✓ Reduced TUNEL±ve	−
Dayer AG et al. 2007 [24]	bFGF	2 weeks	✓ DCX ✓ GFAP, NG2	−	−	Better cortical depth
Jenny B et al. 2009 [25]	bFGF	−	✓ DCX	−	−	−
Jiang XC et al. 2019 [26]	BDNF	−	−	−	−	Accumulated to injury site
Kameda M et al. 2007 [27]	GDNF	NS: 1 week	✓ PSA-NCAM ✓ GFAP	−	✓ Reduced Iba1 ✓ Reduced TUNEL±ve	Maximum 1 mm
Korshunova I et al. 2020 [28]	Bcl2l1	1 week	−	−	−	−
Lee HJ et al. 2007 [29]	VEGF	8 weeks	✓ NF-L/NF-H or MAP2 ✓ GFAP	−	✓ Microvessel proliferation ✓ Reduced TUNEL±ve	−
Lee HJ et al. 2009 [30]	Akt1	8 weeks	✓ NF-H ✓ GFAP	−	−	−
Lee HJ et al. 2010 [31]	BDNF	8 weeks	✓ NF-H or MAP2 ✓ GFAP	−	✓ Microvessel proliferation ✓ Reduced TUNEL±ve ✓ Anti-apoptosis	−
Liu F et al. 2020 [32]	GDNF	−	✓ MAP2	−	✓ Astrocyte endfeet repair	−
Pignataro G et al. 2007 [33]	Adenosine release	−	−	−	−	−
Sakata H et al. 2012 [34]	SOD1	4 weeks	✓ TUJ1 ✓ GFAP	−	✓ Angiogenesis ✓ Reduced TUNEL±ve	−
Song W et al. 2024 [35]	BDNF	−	−	−	✓ Increased survival gene expression (PI3K, Akt)	−
Tang X et al. 2023 [36]	BDNF	−	✓ MAP2 ✓ GFAP	Patch clamping - functionally integrated: 4 weeks	−	−
Tian L et al. 2019 [37]	LIF	−	✓ GFAP	−	✓ White matter remodelling	−
Wakai T et al. 2013 [38]	SOD1	5 weeks	✓ MAP2 ✓ GFAP	−	✓ VEGF expression ✓ Reduced GDNF expression ✓ Neuronal survival	−
Wang J et al. 2015 [39]	Gal-1	2 weeks	✓ Nestin ✓ NeuN or GFAP	−	✓ Microglia M2 phenotype ✓ Elevated MBP staining	−
Wu W et al. 2010 [40]	HIF-1a	5 weeks	✓ NF	−	✓ Factor VIII±ve endothelial cells	−
Wu Z 2015 [41]	GDNF	7 weeks	−	✓ PSD-95 ✓ Syn protein	−	−
Xu P et al. 2019 [42]	BRCA1	4 weeks	−	−	✓ Reduced TUNEL±ve ✓ Reduced GDNF expression ✓ NGF expression	−
Yao Y et al. 2016 [43]	VEGF	−	−	−	✓ VEGF expression	−
Ye Q et al. 2017 [44]	bFGF	24 hours	✓ NeuN ✓ GFAP	−	−	More NSCs at injury site: 24 hours
Zhang JJ et al. 2017 [45]	bFGF	4 weeks	✓ NeuN ✓ GFAP	−	−	NSCs not detected in contralateral hemisphere
Zhang Z et al. 2008 [46]	NT-3	2 weeks	−	−	−	−
Zhu J et al. 2017 [47]	Noggin	−	−	−	✓ Reduced brain water content ✓ Reduced SOD activity ✓ Reduced malondialdehyde ✓ Reduced TUNEL±ve	−
Zhu JM et al. 2011 [48]	BDNF	12 weeks	✓ NF	−	−	−
Zhu Y et al.[49]	VEGF	−	−	−	✓ Reduced brain water content ✓ Reduced GFAP	−

Note: (-) = study did not measure this outcome, ✓ = yes

Abbreviations: Akt, serine/threonine kinase; DCX, doublecortin; GFAP, glial fibrillary acidic protein; Iba1, ionized calcium-binding adaptor molecule 1; MAP2, microtubule-associated protein 2; NeuN, neuronal nuclei; NF, neurofilament; NF-H, neurofilament heavy chain; NF-L, neurofilament light chain; NG2, nerve/glial antigen 2; PI3K, phosphoinositide 3-kinase; PSA-NCAM, polysialylated-neural cell adhesion molecule; PSD 95, postsynaptic density protein 95; SOD, superoxide dismutase; Syn, synuclein; Syp, synaptophysin; TUJ1, beta III-tubulin; TUNEL, Terminal deoxynucleotidyl transferase dUTP Nick End Labelling

Abbreviations for genetic modifications: Akt1, RAC (Rho family)-alpha serine/threonine-protein kinase; BCL2L1, Bcl-2-like protein 1; BDNF, brain-derived neurotrophic factor; bFGF, basic fibroblast growth factor; BRCA1, breast cancer gene 1; Gal-1, galectin 1; GDNF, glial cell line-derived neurotrophic factor; HIF-1a, hypoxia-inducible factor 1-alpha; LIF, leukemia inhibitory factor; NT-3, neurotrophin-3; NGF, nerve growth factor; SOD1, superoxide dismutase 1; Ubc9, SUMO-conjugating enzyme ubiquitin carrier protein 9; VEGF, vascular endothelial growth factor

Thirteen studies reported outcomes for late-period survival (assessed at 28 days or greater post cell administration). All studies found genetically modified NSCs to significantly improve graft survival rates compared to naïve NSCs. All three included studies conducted by Lee HJ et al. found their genetically modified NSCs to survive better than naïve NSCs at 8 weeks; 39% vs. 13.8% (VEGF) [29], 33% vs. 16% (Akt1) [30] and 38.3% vs. 13.2% (BDNF) [31]. Tang X et al. reported their genetically modified NSCs overexpressing BDNF had a survival rate of 48.33% which was significantly higher than their naïve NSC group of approximately 25% [36]. These findings were reflected by the other 9 studies who measured cell counts or immunocytochemistry visual comparisons or expression levels of upregulated target gene. Kameda M et al. (GDNF) also reported significant higher survival rates in the genetically modified NSCs by 28 days post-transplant [27]. These findings demonstrate that a portion of the grafted genetically modified NSCs have the capacity to proliferate and remain at the site of injury for over a month, suggesting therapeutic potential even in the chronic phase of stroke pathology.

### Graft migration

Given most studies utilised intracerebral NSC transplantation we were interested to understand the impact of NSC modification on the migration of NSC when cells were delivered systemically. In total, four studies utilised systemic routes of administration; three being intravenous and one intranasal (Table 3).

Of the four studies, only Jiang XC et al. (BDNF) tracked the migration of NSCs and found that NSCs first appeared in the heart, liver, kidneys, then finally accumulated within the ischaemic region [26]. Both Zhang JJ et al. and Ye Q et al. found that NSCs genetically modified to overexpress bFGF were able to home to the injury site, with few to none in the contralateral hemisphere [44, 45]. Tian L et al. who over-expressed LIF, did not comment on migratory capacity differences between its genetically modified and naïve NSCs [37].

Although the remaining studies all utilised intracerebral transplantation, better migration to the site of injury was still reported for the genetically modified NSCs. Overall, studies found genetically modified NSCs migrated further and increased depth of cortical invasion (Dayer AG et al. (bFGF) [24], Kameda M et al. (GDNF) [27], Lee HJ et al. (Akt1) [30], Zhu J et al. (Noggin) [47]). However, two studies reported limited migratory capacities (Chen B et al. (GDNF) [23] and Wang J et al. (Gal-1) [39]). Furthermore, two studies found no differences between genetically modified and naïve NSCs (Tian L et al. (LIF) [37] and Wu Z (GDNF) [41]) in terms of cell migration capacity.

### Graft differentiation

Of the 29 included studies, 16 studies reported outcomes for graft differentiation (Table 3). Of these, 10 studies compared differentiation differences between genetically modified NSCs and naïve NSCs. Due to significant heterogeneity between studies in the method of examining differentiation and presentation of data, meta-analysis could not be performed. Most reported time-points for analysis were either 1 to 2-weeks post transplantation or 28-days and over post transplantation. Studies that compared genetically modified NSCs to naïve NSCs at the early timepoints found grafted cells in the genetically modified group to maintain a higher proportion of immature cells. Dayer AG et al. (bFGF) [24] reported approximately 50% of grafted cells expressed DCX, an immature neuronal marker, at 2-weeks. Similarly, Jenny B et al. (bFGF) [25] reported their genetically modified NSC group to express DCX (8.2%) more than the naïve NSC group (1.4%) at 1-week post-transplant. Kameda M et al. (GDNF) found most of the graft expressed Nestin, a NSC specific marker and their genetically modified NSC group had significantly less GFAP-positive cells (22% vs. 46.6%) at 1-week [27]. Other studies that did not compare their genetically modified NSCs to a naïve NSC control found similar outcomes. Wang J et al. (Gal-1) reported less than 10% of the transplanted NSCs expressed NeuN (early neuronal marker) or GFAP (astrocyte marker) at 2-weeks [39]. Interestingly, the Lee HJ et al. group reported that their genetically modified graft differentiated into NF-H-positive neurons (35–45%) for all three studies at 2-weeks [29–31].

At later timepoints, most studies observed increased downstream differentiation of grafted NSCs. Five studies found their genetically modified NSCs were able to differentiate into mature neurons at a greater rate than naïve NSCs, as identified with mature neuron markers NeuN and MAP2 (Ye Q et al. (bFGF) [44], Zhang JJ et al. (bFGF) [45], Bernstock JD et al. (Ubc9) [22], Tang X et al. (BDNF) [36]). Two studies that investigated the effect of SOD1 found no differences in graft differentiation composition between genetically modified NSCs and naïve NSCs (Sakata H et al. (Sod1) [34], Wakai T et al. (Sod1) [38]). Interestingly, Wakai T et al. found that most of their grafted cells in both genetically modified and naïve NSCs differentiated into astrocytes [38], and Tian L et al. (LIF) found their genetically modified NSCs group to have significantly higher astrocytes than the naïve NSC group [37]. Overall, these studies demonstrate the capacity of genetically modified NSCs to differentiate into mature neurons, however the functional status of these cells have not been confirmed.

### Impact of genetically modified NSCs on endogenous tissue repair

Of the 29 included studies, 14 studies reported on interactions between graft and endogenous tissue microenvironment (Table 3). Most studies revealed that genetically modified NSCs were able to reduce neuroinflammation to a greater extent than naïve NSCs. Four studies found improvements in angiogenesis; Sakata H et al. (SOD1) [34] found the genetically modified NSCs to have significantly higher blood vessel density; Lee HJ et al. 2007 (VEGF) [29] and 2010 (BDNF) [31] reported significantly higher factor VIII-positive microvessels; and Wu W et al. reported increased factor VIII-positive endothelial cells [40]. Two studies reported genetically modified NSCs to reduce microglial activation (Kameda M et al. (GDNF) [27] and Wang J et al. (Gal-1) [39]). In addition, Wang J et al. found the genetically modified NSCs increased the “M2-like” microglia phenotype [39]. Eight studies reported genetically modified NSCs to reduce apoptosis either through assessment of TUNEL staining, or caspase-3 expression [22, 23, 27, 29, 31, 34, 42, 47].

Four studies reported that genetically modified NSCs were able to enhance endogenous neuronal integration. Bernstock JD et al. (Ubc9) reported increased PSD-95 (postsynaptic density protein 95), a marker for synapses in the post-stroke brain in genetically modified NSC grafted animals at 30 days post cell administration [22]. Chen B et al. (GDNF) reported enhanced Syp (synaptophysin) protein expression in the animals treated with genetically modified NSC compared to naïve NSCs at 2 to 3-weeks following cell administration [23]. Tang X et al. (BDNF) showed that graft derived neurons were able to receive and send synaptic signals to endogenous neurons, generating action potentials and had similar electrophysiological properties to endogenous neurons [36]. Wu Z (GDNF) showed that transplantation of genetically modified NSCs had higher levels of synaptic protein and PSD-95 compared to both naïve NSCs and vehicle/injury control. [41] These findings indicate that genetically modified NSCs have the potential to facilitate neuronal repair and replacement.

Four studies also found that genetically modified NSCs facilitated repair of endogenous tissues. Tian L et al. (LIF) reported the genetically modified NSCs had increased MBP (myelin basic protein) intensity in white matter bundles indicating significantly improved white matter repair [37]. Zhu J et al. (Noggin) reported attenuation of brain water content and MDA (malondialdehyde) content at 28 days post transplantation of genetically modified NSCs, demonstrating their ability to reducing post-stroke oedema [47]. Zhu Y et al. (VEGF) echoed this finding and reported the genetically modified NSCs significantly reduced brain water content, reducing brain oedema [49]. Liu F et al. (GDNF) found genetically modified NSCs led to significantly greater repair of astrocyte end-feet compared to naïve NSCs, indicating better potential for blood brain barrier repair after stroke [32].

#### Risk of bias and publication quality assessment

**Fig. 9:**
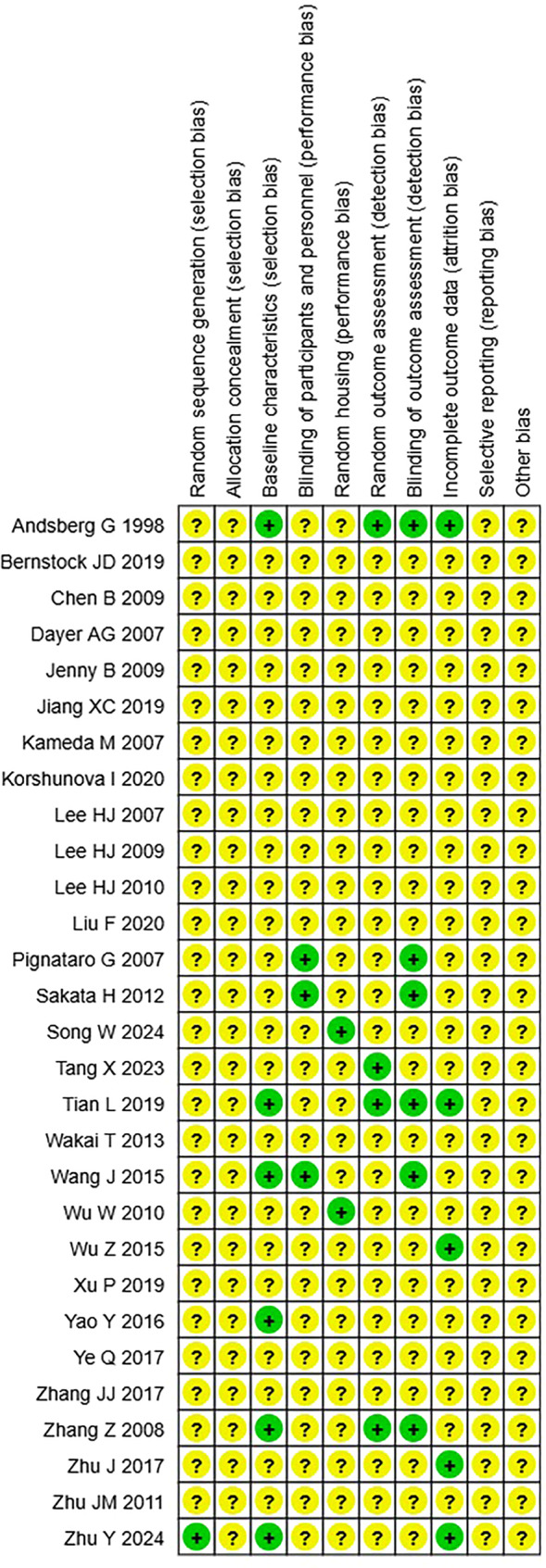
Risk of bias of the included studies. (+) = low risk of bias, (?) = unclear risk of bias, (**x**) = high risk of bias

Risk of bias assessment was conducted using the SYRCLE risk of bias tool, results are summarized in Fig. 9. No studies were judged to have a high risk of bias in any domain; however, no studies obtained a low risk of bias judgement across all domains either. Only one study described random sequence generation methods, and no studies described allocation concealment methods. Low risk of bias was observed in less than half of the included studies in the following domains: baseline characteristics (*n* = 6), blinding of participants and personnel (*n* = 3), random housing (*n* = 2), random outcome assessment (*n* = 4), blinding of outcome assessment (*n* = 6) and incomplete outcome data (*n* = 5). The domains of selective reporting and other bias were all judged unclear given published protocols, and detailed methods were not available.

GRADE assessment rated the certainty of evidence for both infarct volume (Supp. Fig. 14) and neurological functional recovery (Supp. Fig. 15) as very low. Genetically modified NSCs showed large reductions in infarct volume (SMD 4.53 vs. vehicle; 1.07 vs. naïve) and substantial improvements in NSS (SMD 4.28 vs. vehicle; 2.75 vs. naïve), but serious risk of bias, inconsistency, indirectness, imprecision, and suspected publication bias limit confidence in these estimates. Despite the strong observed effects, the findings remain highly uncertain.

## Discussion

Currently there are no neuroprotective or neuroregenerative therapies that have a high evidence level available for stroke patients. While genetically modified NSCs holds promise in amplifying the therapeutic potential of NSCs, many questions remain unanswered. Although there have been a number of studies investigating the efficacy of genetically modified NSCs, the high level of heterogeneity between studies makes it difficult for conclusions to be drawn. The wide range of different genes tested shows the multitude of different pathways NSCs can be enhanced to improve their survival at, homing to and modulation of the ischemic stroke injury site.

Our meta-analysis revealed a greater therapeutic potential of genetically modified NSCs for both lesion volume reduction and improvement in neurological functional recovery compared with both naïve NSC treatment and vehicle/injury control. Our findings suggest that genetically modified NSCs could help overcome efficacy limitations of previous naïve NSC therapy trials. From subgroup analyses, discrepancies between the impact of timing of administration and total cell dose were found between the two primary outcomes of lesion volume reduction and neurological function recovery. Whilst there was a potentially greater effect on lesion volume reduction for cell therapy administered over 24 hours post-injury, there was greater benefit for cell therapy administered at 24 hours or less for neurological function recovery. Similarly, there was a potentially greater effect of total cell dose less than 1 M for lesion volume reduction, contrasted to a slightly greater effect of total cell dose of over 1 M for neurological function recovery. It is important to keep in mind that we observed significant heterogeneity between included studies, especially in terms of treatment protocol utilised. Funnel plot assessment also found high asymmetry, indicating substantial publication bias or small-study effects. Furthermore, GRADE assessment rated the certainty of evidence for both infarct volume and neurological functional recovery as very low Therefore, there may be other confounding factors not accounted for. Moreover, due to the limited number of eligible studies, sub-group analysis especially for cell dose and neurological function recovery was significantly underpowered, with only two studies in total using a cell dose of over 1x10^6^. We also performed sub-group analysis for injury model, route of administration, and selected genetic variations but no sub-group differences were identified.

Unfortunately, in our systematic review we did not find a clear candidate gene that was most commonly used, or appeared to be superior compared the others. The comparison between genes was difficult to conduct because each study chose to report different outcomes, or use different administration routes of timing of administration, limiting comparisons. In Table 4, we have summarised key outcomes measured for the most commonly studied target genes – BDNF, GDNF, bFGF and VEGF. Although the number of studies included are limited and the risk of bias for each of these studies is high, there appears to be some potential trends. All four genes appear to have positive effects for lesion volume reduction and functional recovery. GDNF and bFGF appear to have greater effects for graft differentiation into neurons, whilst only bFGF appears to have positive effects for migratory capacities. Graft NSC survival, integration with endogenous neural circuits, interaction with the micro-environment and anti-inflammatory effects were not commonly reported by studies. This also highlights the lack of potential mechanistic outcomes reported by studies in this field.

**Table 4:** Commonly studied genes; BDNF, GDNF, bFGF and VEGF

	BDNF (n = 5)	GDNF (n = 4)	bFGF (n = 4)	VEGF (n = 3)
**Lesion volume reduction**	✓	✓	✓	✓
	3/3	3/4	3/4	2/3
	2 studies did not report outcomes	1 study did not measure significance	1 study no significance	1 study did not report
**Functional recovery (all behavioural tests)**	✓	✓	?	✓
	4/5	3/3	2/2	2/3
	1 study did not report significance	Significance by 1–3 weeks	2 studies did not measure	1 study no significance
**Graft survival modified cells better than naive**	?	?	?	?
	2/2	2/3	2/2	1/1
	3 studies did not compare	1 study did not find significance	2 studies did not report	2 studies did not report
**Graft differentiation into neurons**	?	✓	✓	**X**
	1/3	2/2	4/4	0/1
	2 studies did not compare	2 studies did not report outcomes	NeuN or DCX	More astrocytes than neurons
**Endogenous neural integration**	?	✓	N/A	N/A
	1/1	2/2	N/A	N/A
	Integration by 4 weeks	Synaptic protein by 2–3 weeks	Not reported	Not reported
**Anti-inflammatory**	?	?	N/A	✓
	2/2	1/1	N/A	2/2
	Anti-apoptosis, angiogenesis	Reduced microglial activation	Not reported	Angiogenesis, anti-apoptosis
**Migratory Capacities**	**X**	**X**	✓	**X**
	1/3	1/2	3/3	0/1
	Not compared to naïve	1 study no difference	2 IV studies	Significance not measured

Note: ✓ = over 50% studies reported positive outcomes,? = less than 50% studies reported positive outcomes, X = over 50% studies reported negative outcomes. Abbreviations: DCX, doublecortin; IV, intravenous; NeuN, neuronal nuclei

An exciting finding across the studies was the potential impact of increased graft survival on lesion volume reduction. Of the 17 studies that reported graft survival outcomes, 10 studies also reported outcomes on lesion volume reductions. Of the 10 studies that reported both outcomes, eight studies reported genetically modified NSCs had significantly greater reduction in lesion volume compared to naïve NSCs. However, 2 studies did not find statistically significant differences between genetically modified and naïve NSCs (Zhang JJ - bFGF, Bernstock JD – Ubc9) [22, 45]. There was a smaller overlap of studies that measured both graft survival and tested neurological function. Overall, there appears to be a trend of improved lesion volume reduction with better graft NSC survival. However, conclusions cannot be made for functional recovery due to the lack of studies that reported on graft NSC survival. Although it is difficult to ascertain whether there is a direct correlation between higher NSC graft survival and improvement in lesion volume reduction and functional recovery, the relationship aligns with current theoretical expectations. Since genetically modified NSCs can express selected target genes while exerting established neuroprotective effects such as increasing anti-inflammatory activities, having more of the cells present should result in a greater neuroprotective effect.

Another interesting association is the effect that increased NSC differentiation may have on lesion volume reduction and enhanced functional recovery. There were only eight studies in our systematic review that reported on both outcomes for lesion volume reduction and/or neurological functional recovery. Unfortunately, there did not appear to be any associations between favouring any downstream differentiation pathway and improved lesion volume reduction and/or neurological functional recovery. Two studies reported genetically modified NSCs to both have better lesion volume reduction and neurological functional recovery; Tian L et al. (LIF) reported significantly higher graft differentiation into astrocytes [37], whereas Tang X et al. (BDNF) reported significantly higher graft differentiation into mature neurons [36]. Of the studies that found better lesion volume reduction, Sakata H et al. (SOD1) and Wakai T et al. (SOD1) did not find significant differences in genetically modified NSC downstream differentiation [34, 38], whereas Kameda M et al. (GDNF) found genetically modified NSCs to have significantly less astrocyte differentiation [27], and Ye Q et al. (bFGF) found genetically modified to have significantly more neuronal differentiation [44]. Interestingly, Bernstock JD et al. (Ubc9) found no statistically significant differences in lesion volume reduction between genetically modified and naïve NSCs, and also found no significant differences in graft downstream differentiation [22].

Notably, a few studies found that the expression of upregulated target gene actually decreased overtime. Dayer AG et al. reported that by 2 weeks post-transplantation, only 31% of grafted NSCs still expressed bFGF, and hypothesised this to be correlated with graft differentiation [24]. This finding was echoed by Zhu JM et al [48]. While they did not directly compare graft survival outcomes to naïve NSCs, they found that by 1-week post-transplantation, only 25% of grafted NSCs continued to express BDNF [48]. Similarly, Zhang Z et al. found that secretion of NT-3 expression in NSCs halved by 2-weeks post-transplantation [46]. It is not understood what is driving this reduction in expression over time, but it does warrant further investigation.

### Insights from subgroup analysis

Our findings further highlight the difficulty of ascertaining an optimal protocol for stem cell therapy for stroke. Key aspects such as timing of administration, total cell dose and route of administration have not shown consistent results across existing literature. While we tried to conduct sub-group analysis, our results must be interpreted with caution as there were only a limited number of studies that could be included and compared.

#### Timing of cell administration

Clinically, stroke patients are typically treated with thrombolysis agents or mechanical thrombectomy within 24 hours [50, 51]. However, as shown in Fig. 2, most preclinical studies administered therapy after 24 hours. This could be limited by the route of administration chosen. Since most included studies that transplanted NSCs via an intracerebral route, one main reason for the timing most commonly being delayed until 72 hours, could have been to allow animals time to recover from the surgery to induce the injury before being anesthetised again for intracerebral NSC treatment.

#### Cell dose

This dilemma of significant heterogeneity is also reflected in the wide range of cell doses utilised. Due to the limited number of studies that could be included in the meta-analysis, our subgroup analysis was underpowered given only three studies that reported lesion volume reduction outcomes utilised a total cell dose of over 1x10^6^ and only two studies for neurological function recovery. The lack of studies utilising higher doses may have been attributed to the predominant use of intracerebral transplantation as the preferred route of administration for modified NSC stroke treatment, as high doses using this route may result in increased risk of further brain injury such as causing microembolism and increased shear forces damaging graft cells [52, 53].

#### Route of administration

Sub-group analysis of routes of administration, did not find any significant sub-group differences, but there was a small number of studies to compare, publication bias was detected, and the quality of the evidence was low. This is a significant knowledge gap that remains, as intracerebral administration may be much more difficult for clinical translation given very few stroke patients require cranial surgery for acute treatment. Thrombolytic drugs are administered intravenously and mechanical thrombectomy is conducted through intra-arterial stent retrievers [54]. Both methods would be easier to incorporate into routine clinical practice compared with intracerebral transplantation.

#### Limitations

A major limitation of this review is the high heterogeneity across studies, combined with the common lack of reporting transparency in preclinical research, which collectively contribute to the overall low quality of the evidence. Consequently, these factors limit the confidence in the findings and highlight the need for more rigorous and standardized future studies. Through our systematic review and meta-analysis, we sought to specifically compare the efficacy of genetically modified NSCs for stroke and excluded other methods of NSC enhancements such as preconditioning, tissue scaffolding and microRNAs. This decision was made to limit the number of variables and confounding factors. We acknowledge that this study is limited by the high level of heterogeneity between included studies and the high level of risk of bias which is an inherent issue in preclinical animal studies. Overall, this highlights the need for standardisation of the field in terms of outcomes reported to allow future systematic reviews to make meaningful comparisons.

## Conclusions and future directions

Currently, neuroprotective therapies that target the underlying brain injury following stroke do not exist. While stem cell therapy has been considered a promising domain for decades, there is still a profound amount of knowledge gaps in the field. Our systematic review demonstrates the potential for improved efficacy by using genetically modified NSCs to enhance their efficacy compared to naïve NSCs. Through interrogating existing preclinical research to date, we have identified a few barriers preventing the field from progressing. Firstly, the lack of uniformity in outcomes measured was found to be a significant barrier for cross-study comparisons. Secondly, the lack of an optimised protocol for cell therapy, such as timing of administration and cell dosage increases the number of potential confounding factors for reported results. Thirdly, to date, there has not been any large animal model studies performed that could help further clarify the above variables and move this work a step closer to clinical translation. Finally, neural stem cell therapy should be designed with the target stroke patient population in mind. Commonly, elderly patients with large numbers of co-morbidities are at higher risk of stroke. Therefore, as an adjuvant therapy with a neuroprotective goal, stem cell therapy should be designed to better match existing clinical treatment workflow, most likely through less invasive delivery routes, and ensure treatment risk is kept as low as possible.

Genetic modification of NSCs could be a promising avenue for enhancing the therapeutic benefits of NSCs. However, current literature is highly heterogenous with multiple proteins of interest being investigated with no clear consensus and as such, translation based on current preclinical work is challenging. Based on existing evidence, we could not identify any specific target gene to be the most promising candidate. With further carefully designed preclinical and translational studies that align with clinical workflow, genetically modified NSCs may hold potential as an adjunct neuroprotective therapy in stroke, but substantial research is still needed to establish their efficacy and safety.

## Electronic supplementary material

Below is the link to the electronic supplementary material.

Supplementary Material 1

## Data Availability

The datasets used and/or analysed during the current study are available from the corresponding author on reasonable request.

## Abbreviations

Akt1: RAC (Rho family)-alpha serine/threonine-protein kinase
BCL2L1: Bcl-2-like protein 1
BDNF: Brain-derived neurotrophic factor
bFGF: Basic fibroblast growth factor
BRCA1: Breast cancer gene 1
CI: Confidence interval
DALY: Disability adjusted life years
DARPP-32: Dopamine and cAMP-regulated phosphoprotein
DCX: Doublecortin
ESC: Embryonic stem cell
Gal-1: Galectin 1
GDNF: Glial cell line-derived neurotrophic factor
GFAP: Glial fibrillary acidic protein
H&E: Hematoxylin and eosin
HIE: Hypoxic-ischemic encephalopathy
HIF-1a: Hypoxia-inducible factor 1-alpha
Iba1: Ionized calcium-binding adaptor molecule 1
ICH: Intracerebral haemorrhage
IV: Intravenous
LIF: Leukemia inhibitory factor
MAP2: Microtubule-associated protein 2
MBP: Myelin basic protein
MCAO: Middle cerebral artery occlusion
mNSS: Modified neurological severity score
MRI: Magnetic resonance imaging
NeuN: Neuronal nuclei
NF: Neurofilament
NF-H: Neurofilament heavy chain
NF-L: Neurofilament light chain
NG2: Nerve/glial antigen 2
NGF: Nerve growth factor
NSC: Neural stem cell
NSS: Neurological severity score
NT-3: Neurotrophin-3
PI3K: Phosphoinositide 3-kinase
PSA-NCAM: Polysialylated neural cell adhesion molecule
PSD-95: Postsynaptic density protein 95
SGZ: Subgranular zone
SMD: Standardised mean difference
SOD: Superoxide dismutase
SOD1: Superoxide dismutase 1
SVZ: Subventricular zone
Syn: Synuclein
Syp: Synaptophysin
TTC: 2,3,5-Triphenyltetrazolium chloride
TUJ1: Beta III-tubulin
TUNEL: Terminal deoxynucleotidyl transferase dUTP Nick End Labelling
Ubc9: SUMO-conjugating enzyme ubiquitin carrier protein 9
VEGF: Vascular endothelial growth factor
