# Supplementary material for: Therapeutic efficacy of genetically engineered neural stem cells in cerebral ischemia: a systematic review and meta-analysis
Source: J Transl Med. 2026 Feb 19;24:421. doi: 10.1186/s12967-025-07603-y (PMC13020030; doi:10.1186/s12967-025-07603-y)
Supplement: Supplementary file 1 — Supplementary Material 1 [file 12967_2025_7603_MOESM1_ESM.docx]

**Supplementary Information and Figures**

**Search strategy**

1. Cell type

1. Neural Stem Cell*

2. Neural Progenitor Cell*

3. Neural Precursor Cell*

4. NSC*

5. iPSC-NSC*

6. induced pluripotent stem cell derived-neural stem cells

7. induced pluripotent stem cell derived-neural progenitor cells

2. Intervention

1. Gene* modifi*

2. Gene* engineer*

3. Gene* edit*

3. Condition

1. Stroke

2. Brain injur*

3. Neurological

4. cerebral ischemi*

5. Brain ischemia

6. HIE

7. Hypoxia-ischemia

8. Cerebral hemorrhage

9. Hemorrhagic stroke

10. Intracerebral hemorrhage

Search = Cell type (1 or 2 or 3 or 4 or 5 or 6 or 7) AND Intervention (1 or 2 or 3) AND Condition (1 or 2 or 3 or 4 or 5 or 6 or 7 or 8 or 9 or 10)

Filter excluding review and systematic review.

No time limits.


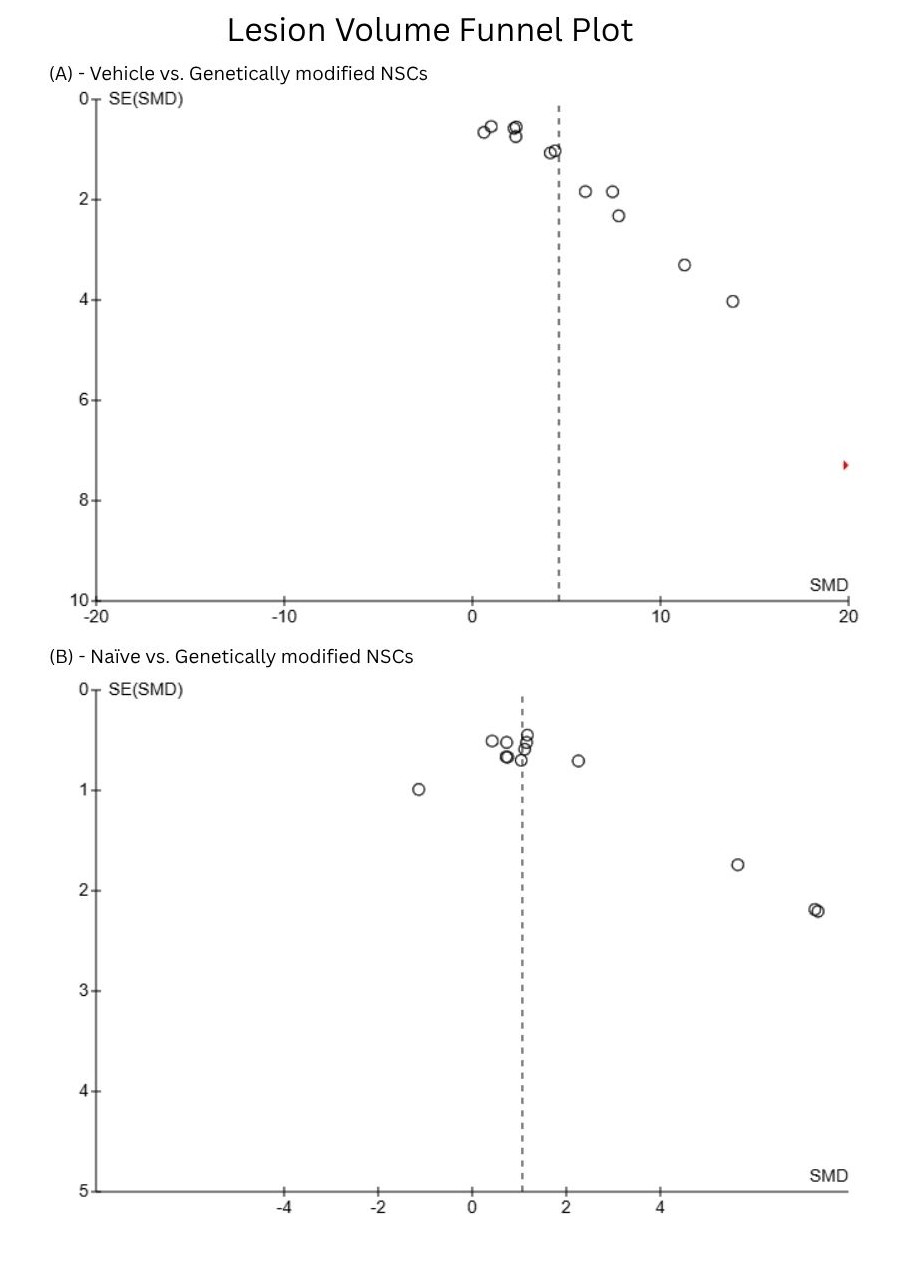


**Supplementary Figure 1. Funnel plots for Lesion Volume meta-analysis**. (A) Vehicle vs genetically modified NSCs. (B) Naïve NSCs vs genetically modified NSCs.


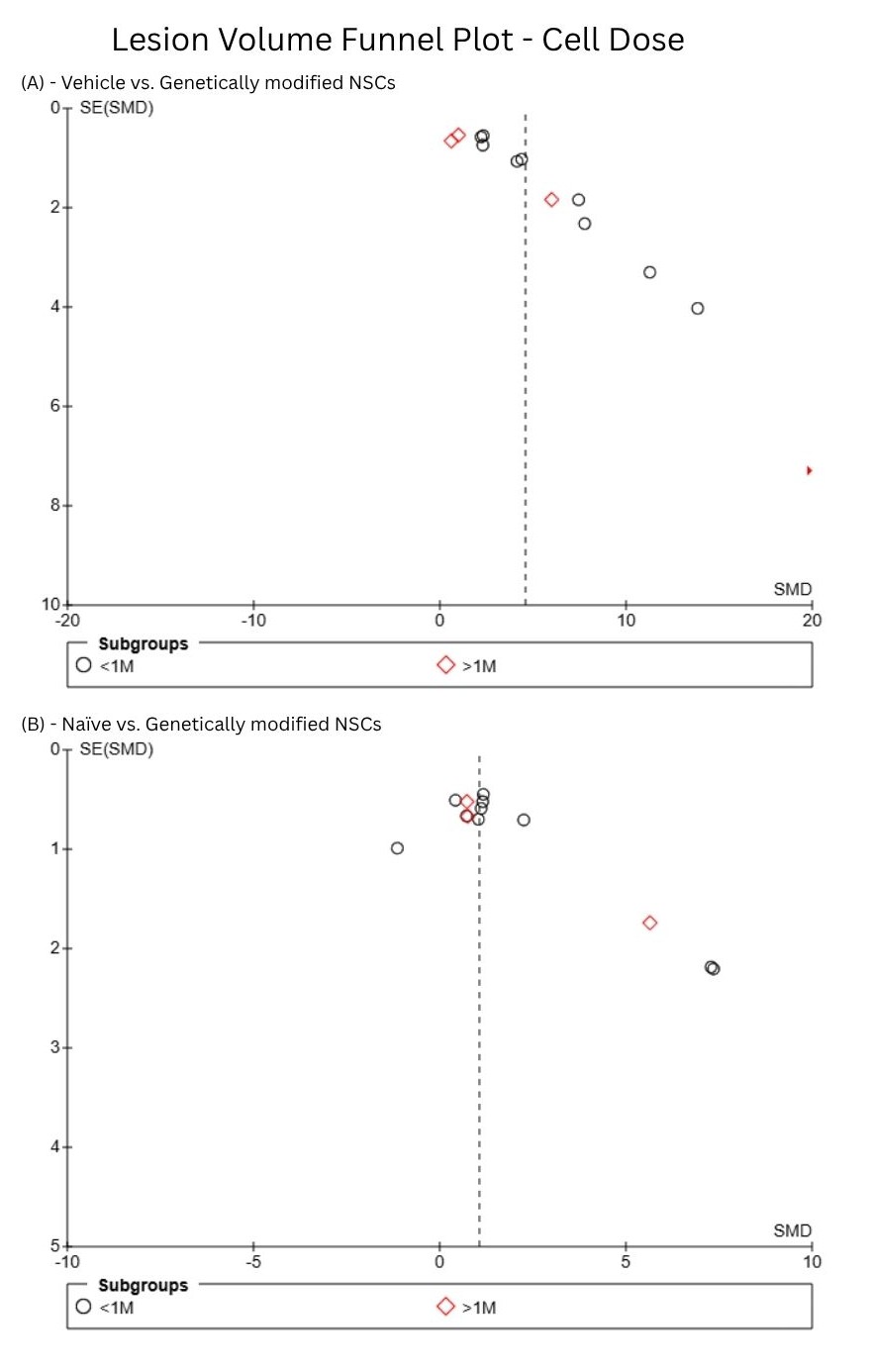


**Supplementary Figure 2. Funnel plots for sub-group analysis of the effect of cell dose on lesion volume**. (A) Vehicle vs genetically modified NSCs. (B) Naïve NSCs vs genetically modified NSCs.


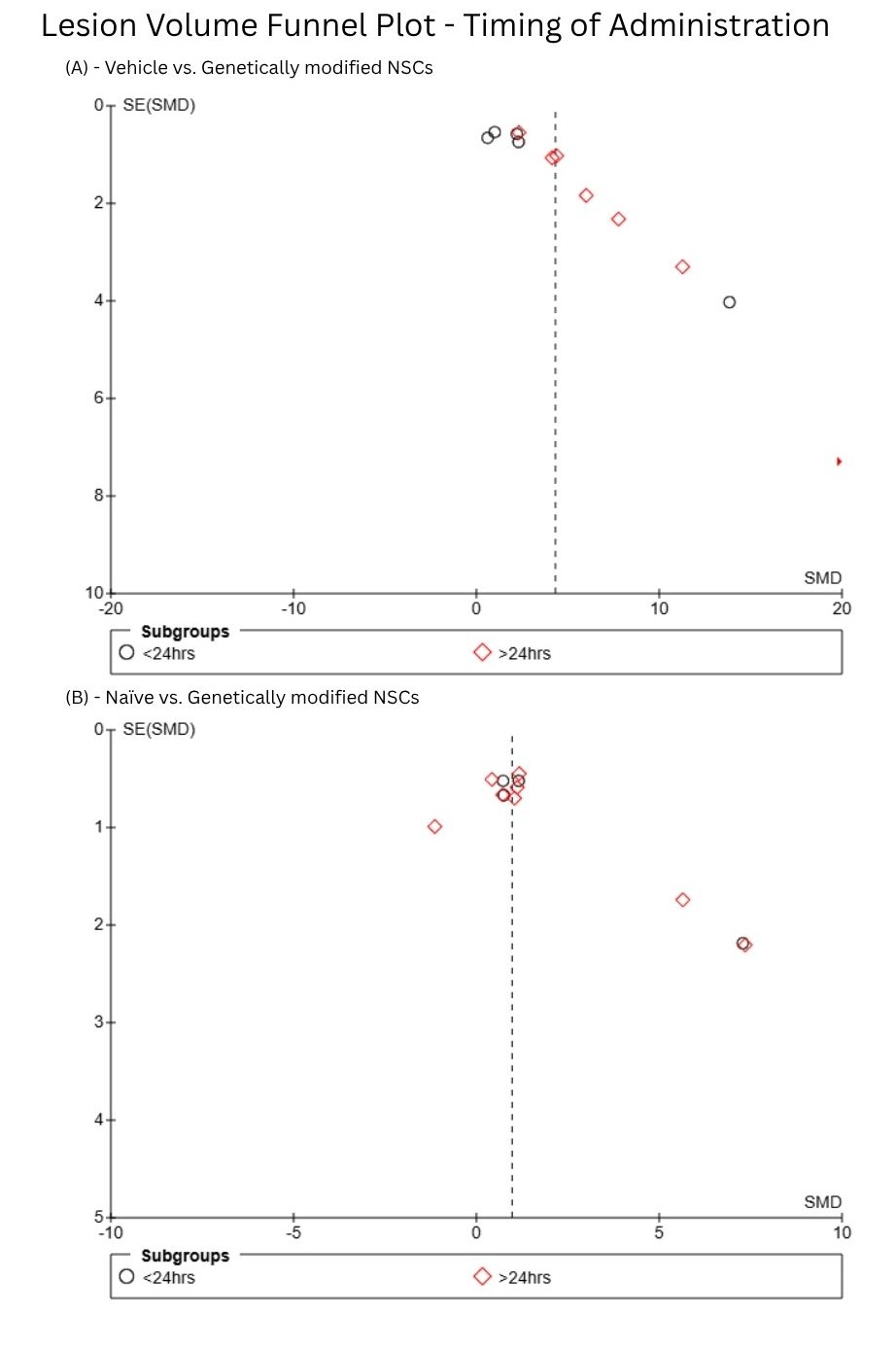


**Supplementary Figure 3. Funnel plots for sub-group analysis of the effect of cell administration timing on lesion volume**. (A) Vehicle vs genetically modified NSCs. (B) Naïve NSCs vs genetically modified NSCs.


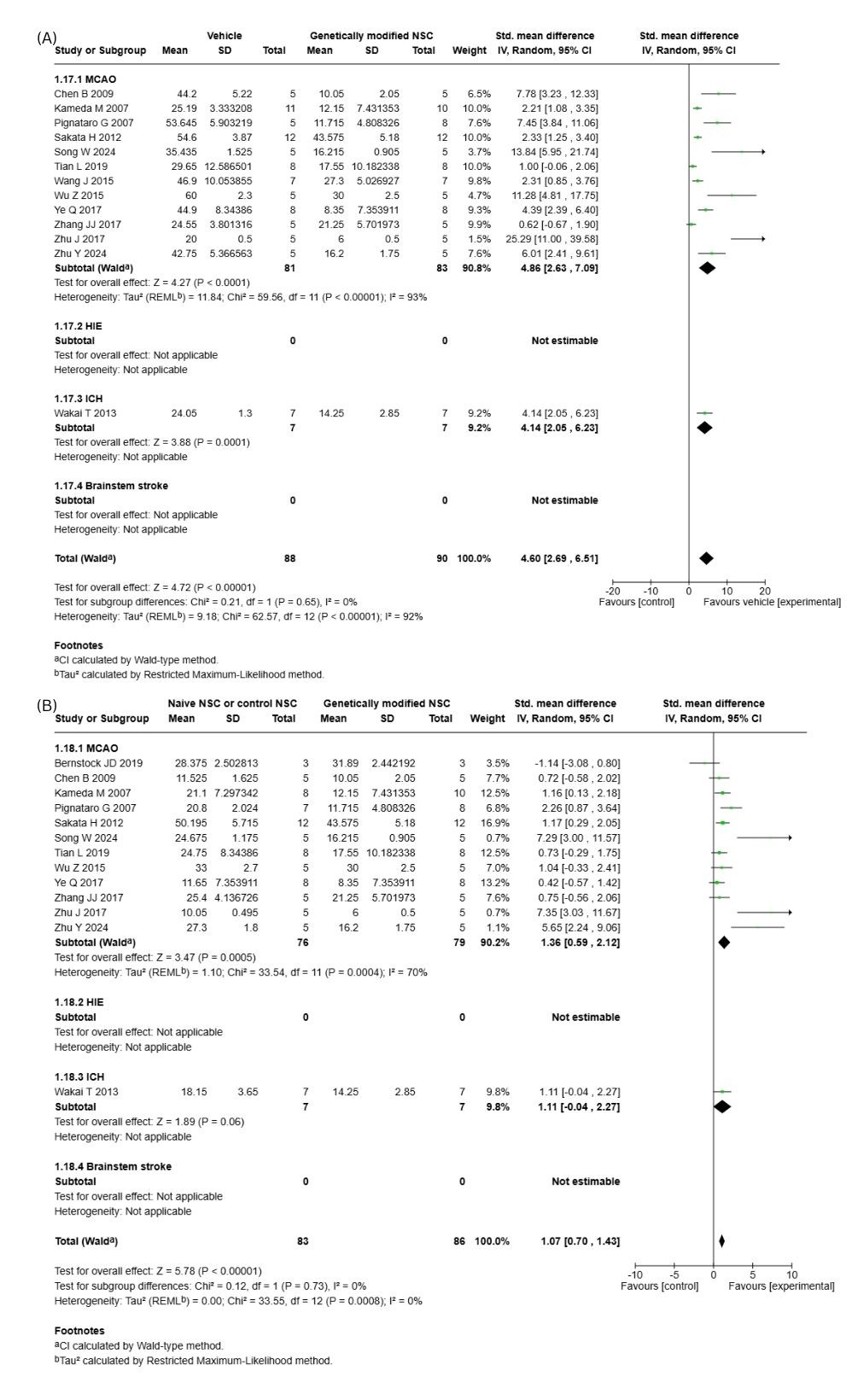


**Supplementary Figure 4. Subgroup Analysis for Lesion Volume Reduction – Type of injury model used.** (A) Vehicle vs genetically modified NSCs. (B) Naïve NSCs vs genetically modified NSCs.


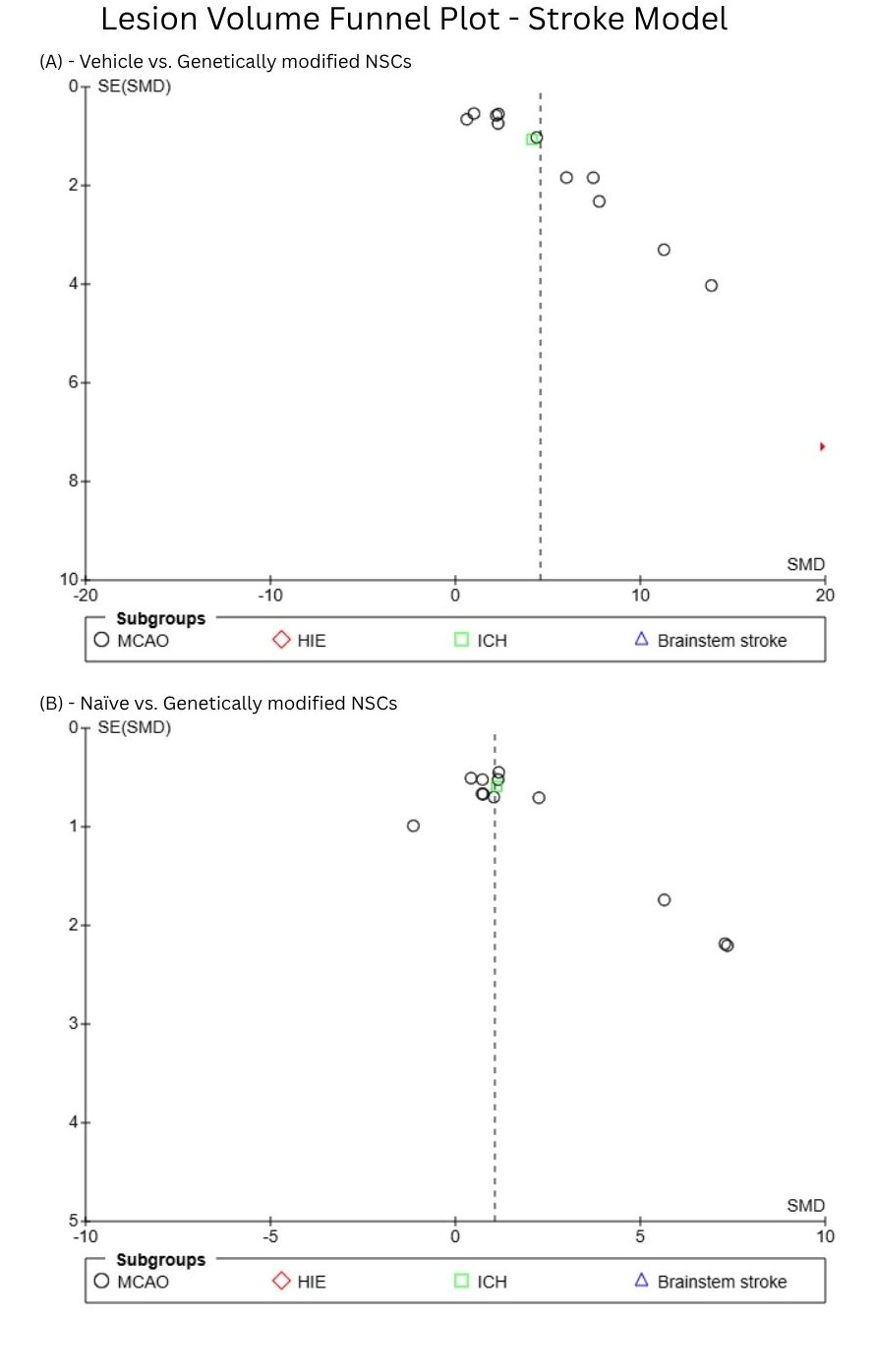


**Supplementary Figure 5. Funnel plots for sub-group analysis of the effect of injury model used on lesion volume**. (A) Vehicle vs genetically modified NSCs. (B) Naïve NSCs vs genetically modified NSCs.


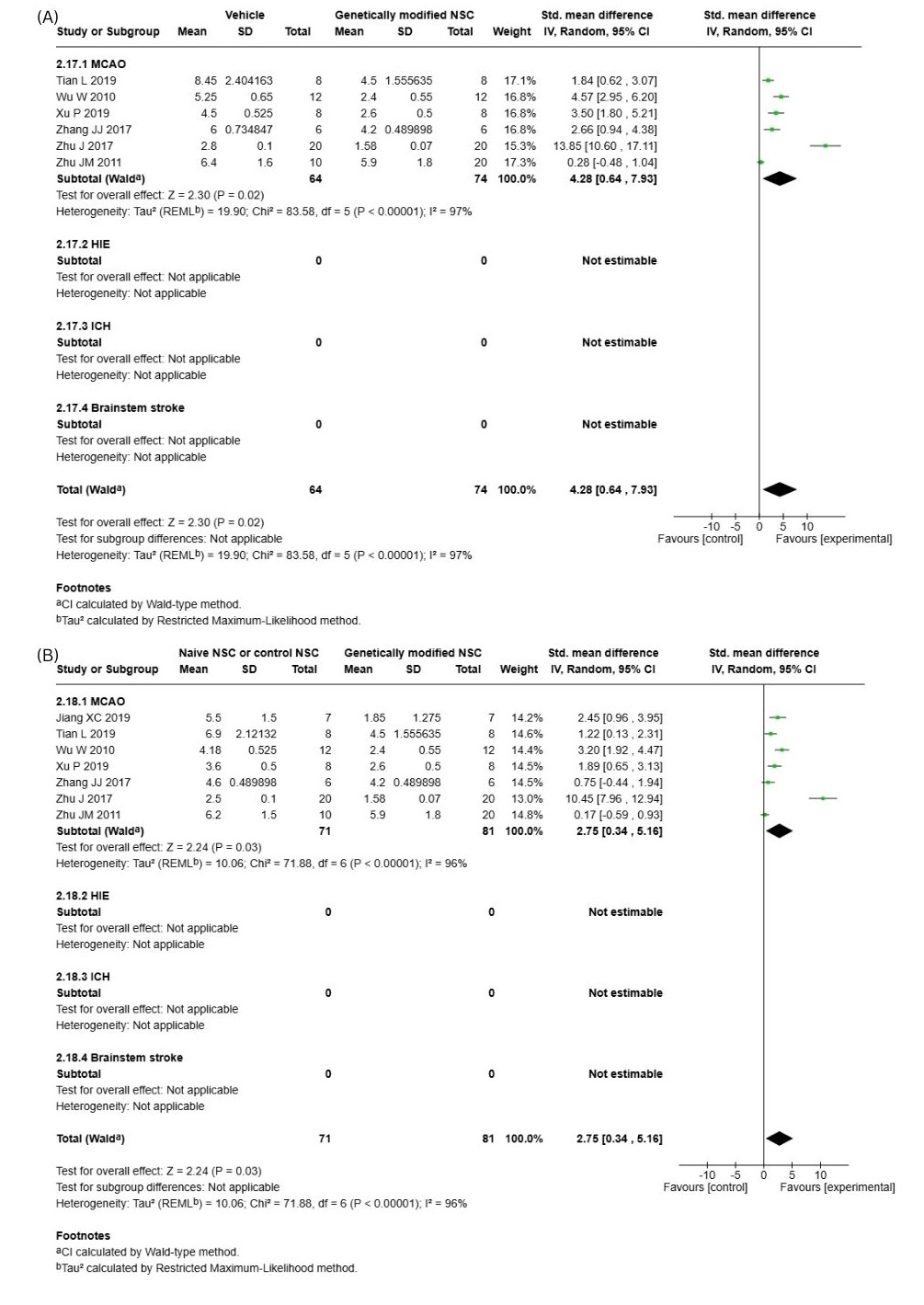


**Supplementary Figure 6. Subgroup Analysis for Neurological Functional Recovery – Type of injury model used.** (A) Vehicle vs genetically modified NSCs. (B) Naïve NSCs vs genetically modified NSCs.


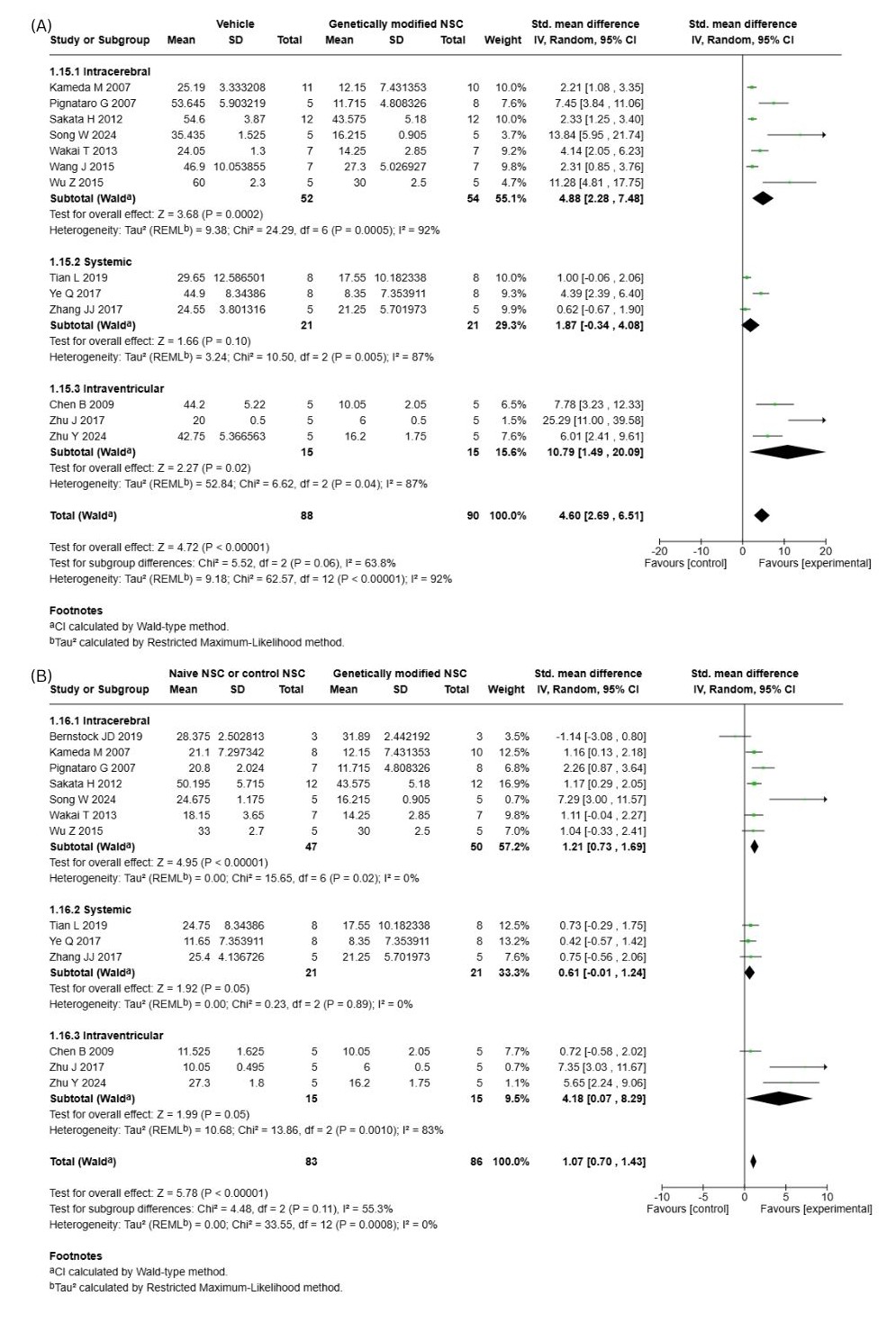


**Supplementary Figure 7. Subgroup Analysis for Lesion Volume Reduction – Route of administration.** (A) Vehicle vs genetically modified NSCs. (B) Naïve NSCs vs genetically modified NSCs.


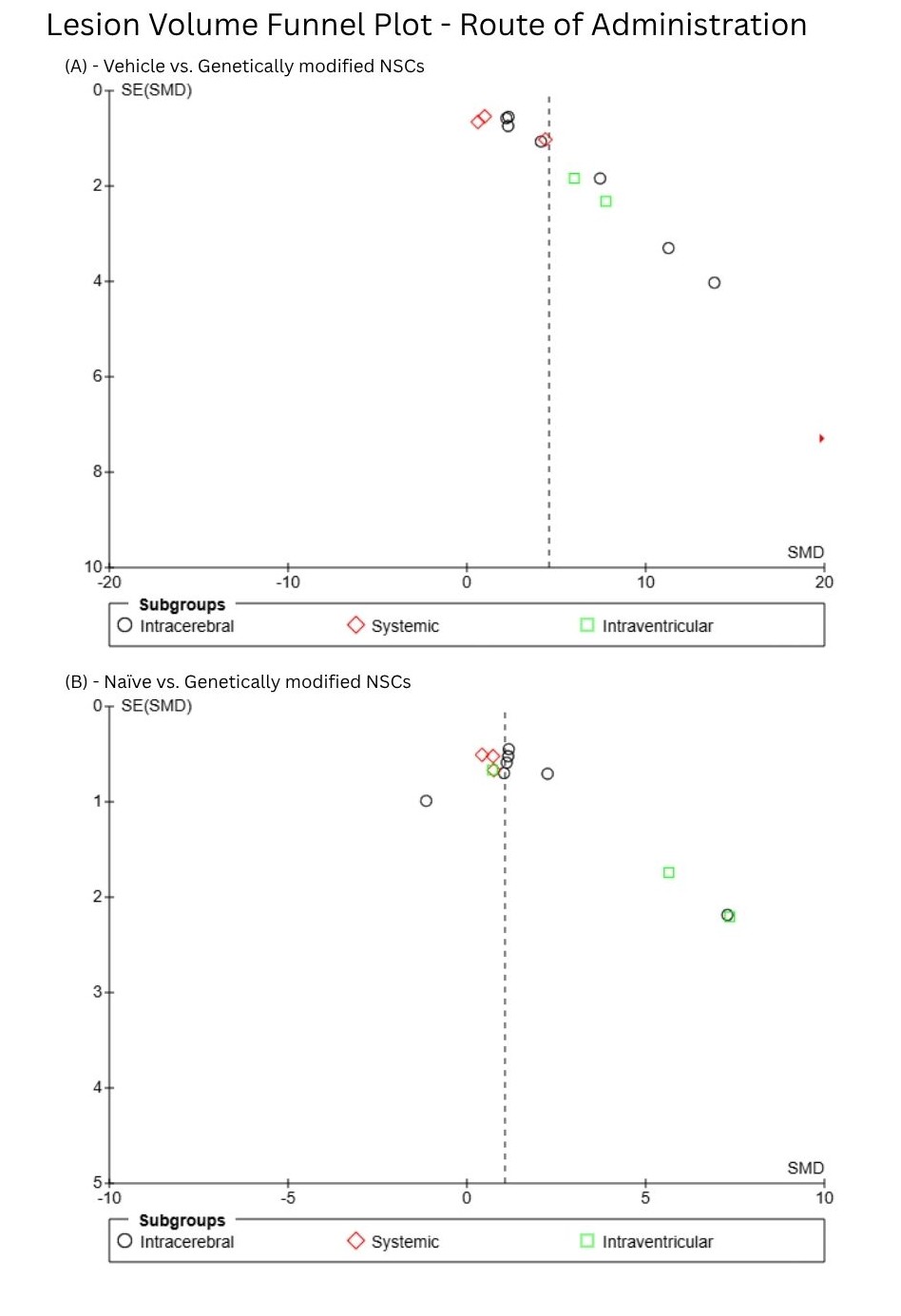


**Supplementary Figure 8. Funnel plots for sub-group analysis of the effect of route of administration on lesion volume**. (A) Vehicle vs genetically modified NSCs. (B) Naïve NSCs vs genetically modified NSCs.


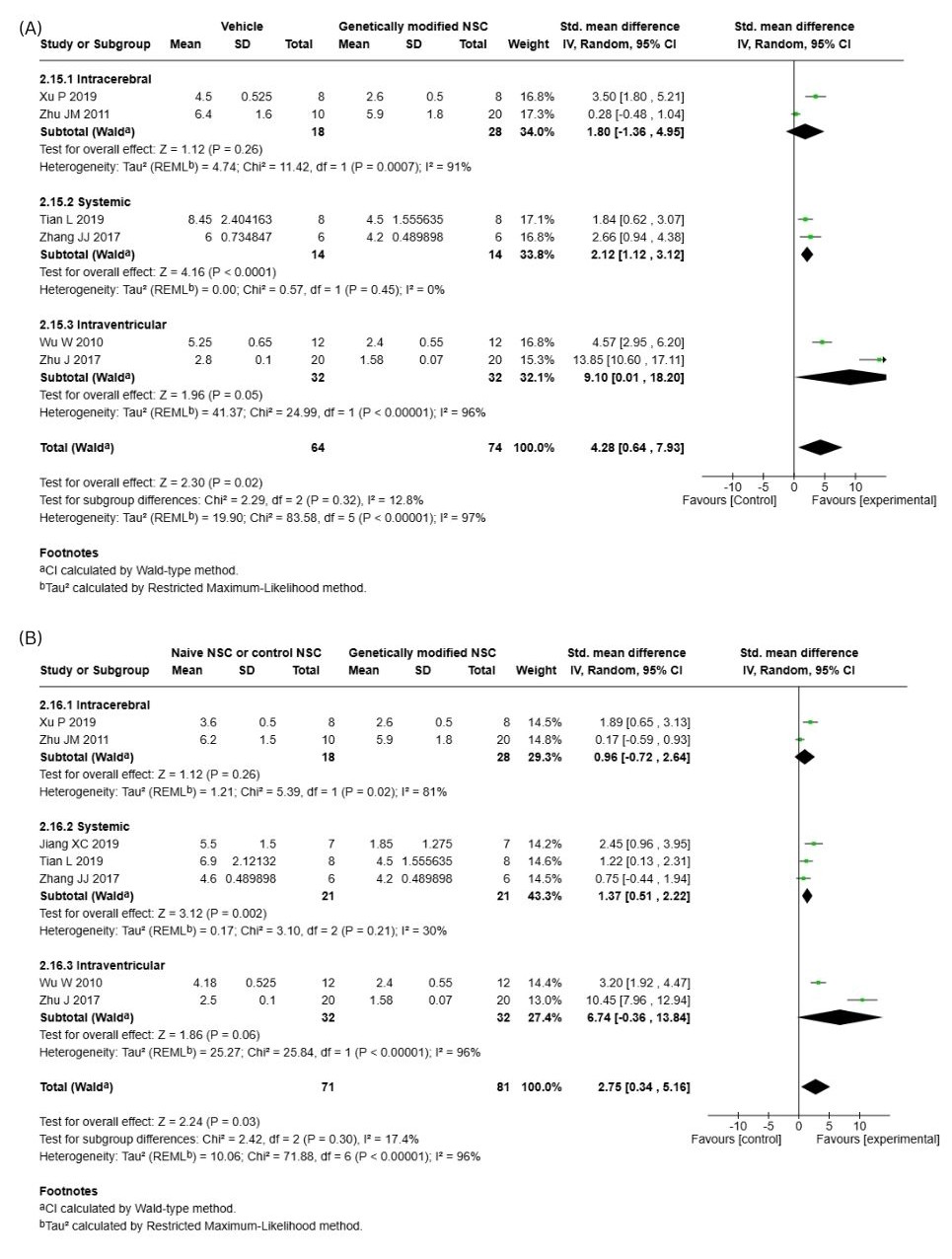


**Supplementary Figure 9. Subgroup Analysis for Neurological Functional Recovery – Route of Administration.** (A) Vehicle vs genetically modified NSCs. (B) Naïve NSCs vs genetically modified NSCs.


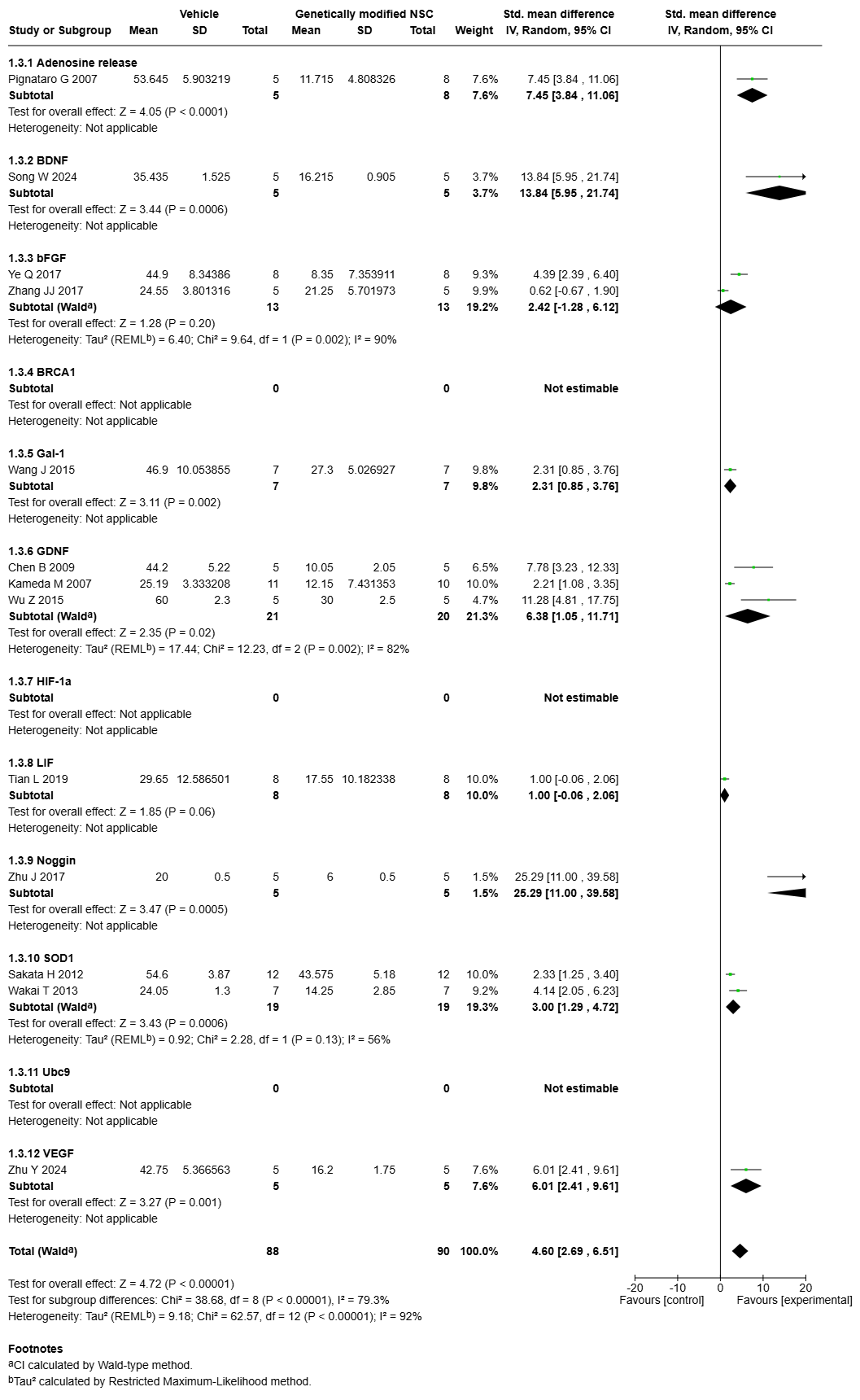


**Supplementary Figure 10. Subgroup Analysis for Lesion Volume Reduction – Specific genetic variation used for vehicle vs genetically modified NSCs.**


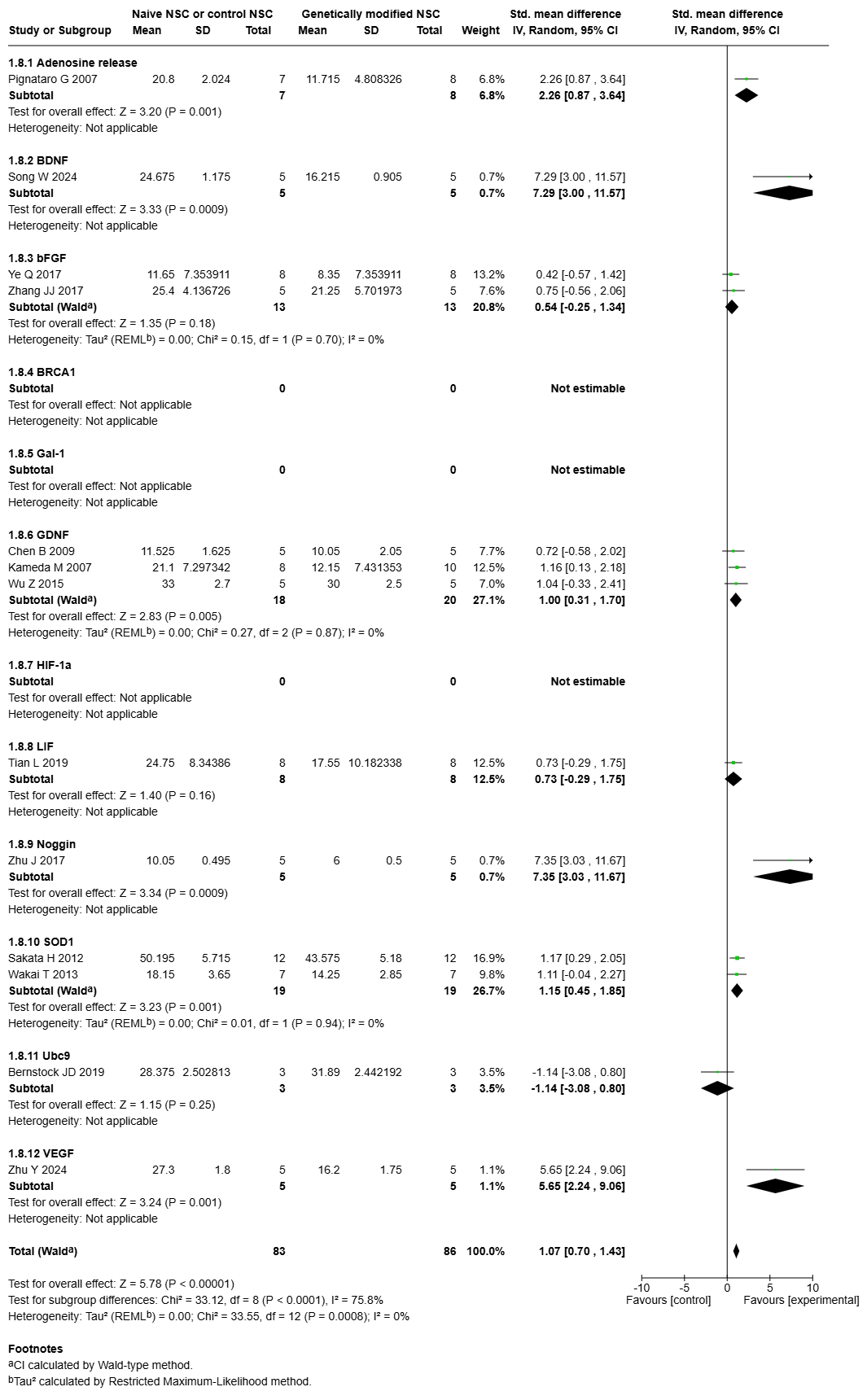


**Supplementary Figure 11. Subgroup Analysis for Lesion Volume Reduction – Specific genetic variation used for naïve NSCs vs genetically modified NSCs.**


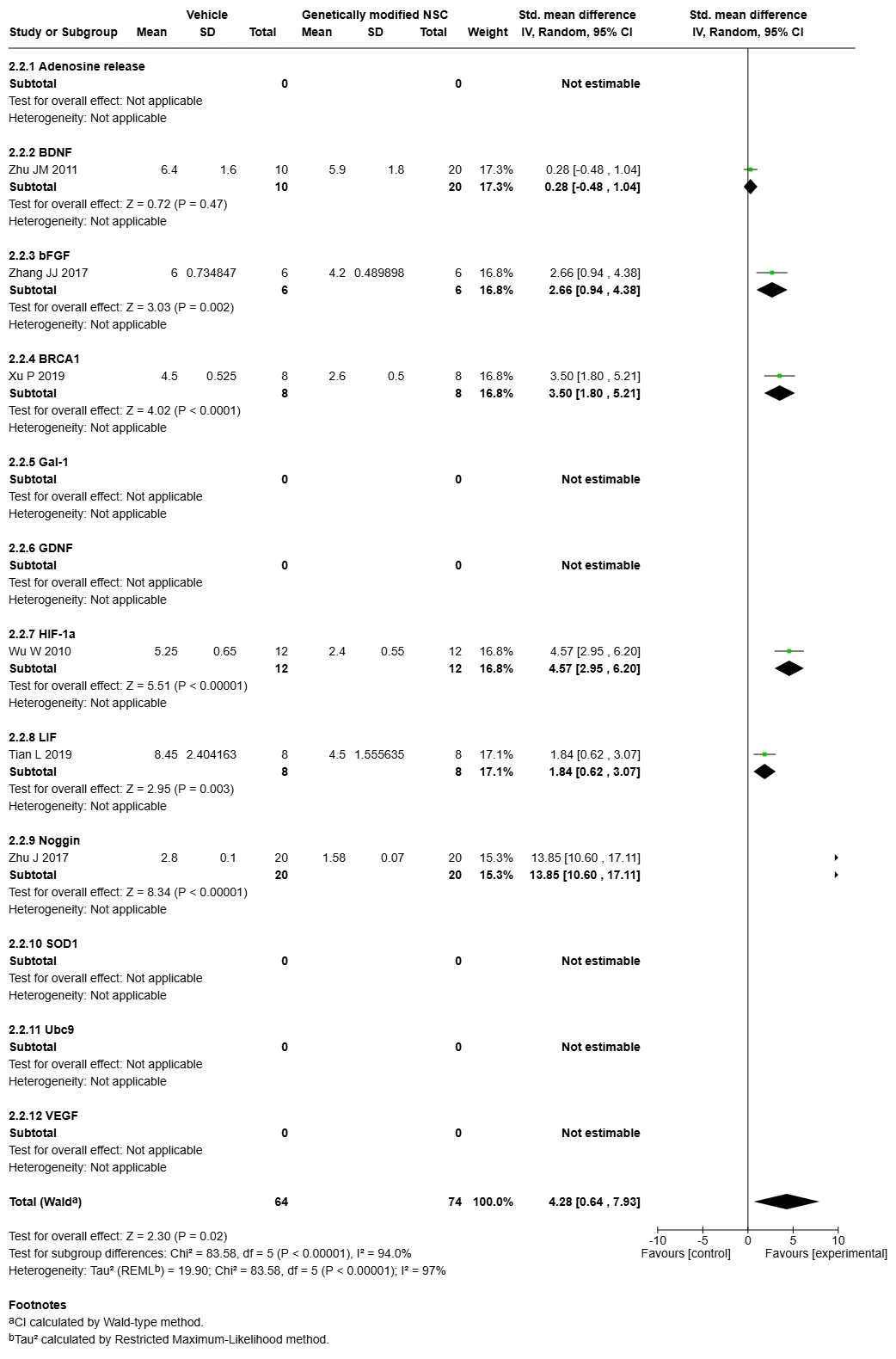


**Supplementary Figure 12. Subgroup Analysis for Neurological Functional Recovery – Specific genetic variation used for vehicle vs genetically modified NSCs.**


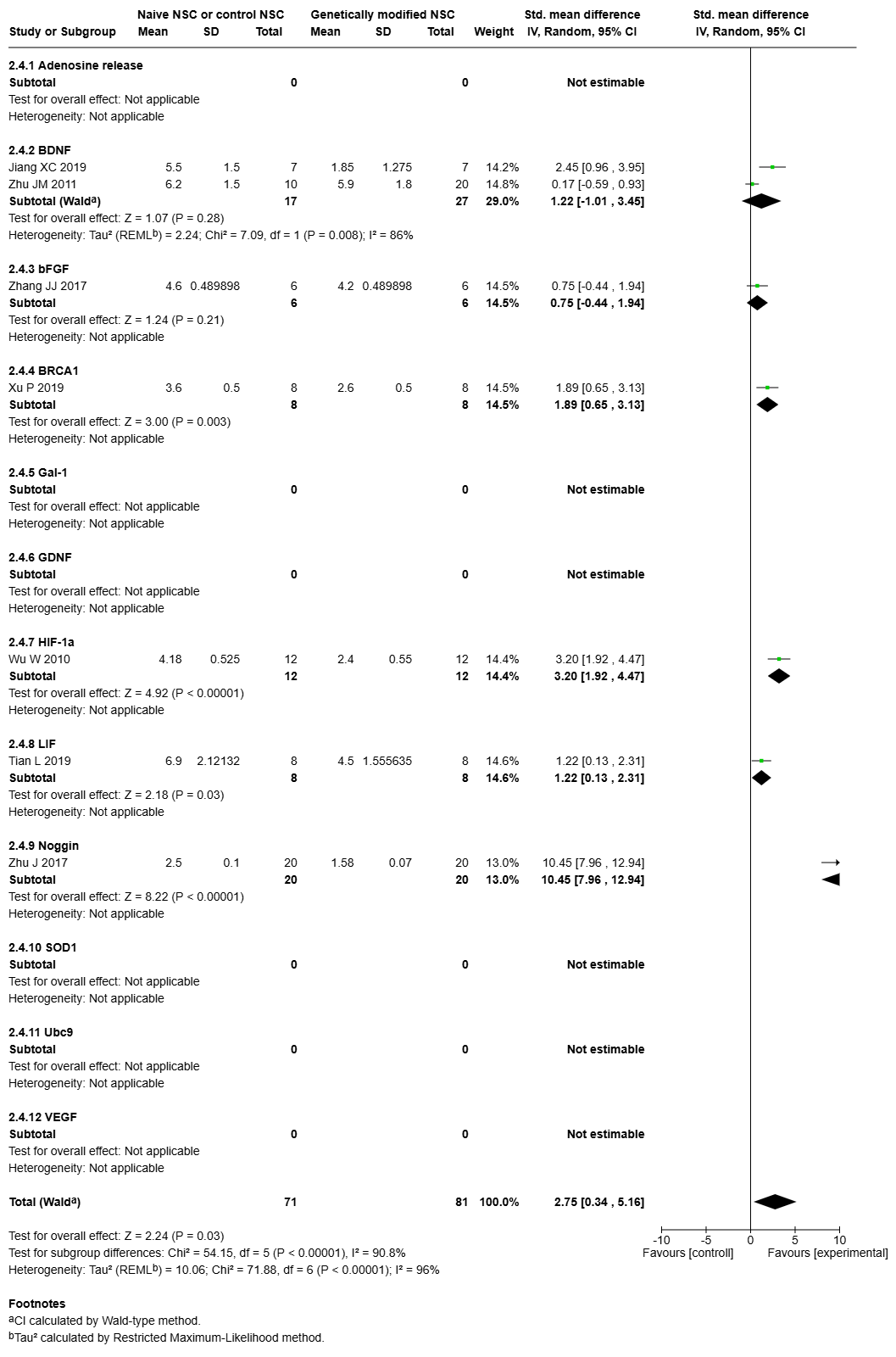


**Supplementary Figure 13. Subgroup Analysis for Neurological Functional Recovery – Specific genetic variation used for naïve NSCs vs genetically modified NSCs.**

| **Certainty assessment** | | | | | | | **№ of patients** | | **Effect** | | **Certainty** | **Importance** |
| --- | --- | --- | --- | --- | --- | --- | --- | --- | --- | --- | --- | --- |
| **№ of studies** | **Study design** | **Risk of bias** | **Inconsistency** | **Indirectness** | **Imprecision** | **Other considerations** | **Infarct Volume** | **[placebo]** | **Relative (95% CI)** | **Absolute (95% CI)** |  |  |
| **Vehicle/Injury vs. Genetically modified NSCs** | | | | | | | | | | | | |
| 13 | non-randomised studies | very serious^a^ | very serious^b^ | serious^c^ | not serious | publication bias strongly suspected very strong association^d^ | 88 | 90 | - | SMD **4.53 higher** (2.66 higher to 6.39 higher) | ⨁◯◯◯ Very low^a,b,c,d^ |  |
| **Genetically modified NSCs vs. Naive NSCs** | | | | | | | | | | | | |
| 13 | non-randomised studies | very serious^a^ | not serious | serious^c^ | serious^e^ | strong association | 83 | 86 | - | SMD **1.07 higher** (0.7 higher to 1.43 higher) | ⨁◯◯◯ Very low^a,c,e^ |  |

**CI:** confidence interval; **SMD:** standardised mean difference

#### Explanations

a. Very serious study design limitations

b. Considerable heterogeneity

c. Differences in cell dose and outcome assessment

d. Asymetry in funnel plot

e. Imprecision

**Supplementary Figure 14. GRADE assessment for Lesion Volume Reduction.**

| **Certainty assessment** | | | | | | | **№ of patients** | | **Effect** | | **Certainty** | **Importance** |
| --- | --- | --- | --- | --- | --- | --- | --- | --- | --- | --- | --- | --- |
| **№ of studies** | **Study design** | **Risk of bias** | **Inconsistency** | **Indirectness** | **Imprecision** | **Other considerations** | **NSS** | **[placebo]** | **Relative (95% CI)** | **Absolute (95% CI)** |  |  |
| **Injury/Vehicle vs. Genetically modified NSCs** | | | | | | | | | | | | |
| 6 | non-randomised studies | very serious^a^ | very serious^b^ | serious^c^ | serious^d^ | very strong association | 64 | 74 | - | SMD **4.28 higher** (0.64 higher to 7.93 higher) | ⨁◯◯◯ Very low^a,b,c,d,e^ |  |
| **Genetically modified NSCs vs. Naive NSCs** | | | | | | | | | | | | |
| 7 | non-randomised studies | very serious | very serious | serious | serious | very strong association | 71 | 81 | - | SMD **2.75 higher** (0.34 higher to 5.16 higher) | ⨁◯◯◯ Very low |  |

**CI:** confidence interval; **SMD:** standardised mean difference

#### Explanations

a. Very serious study design limitations

b. Considerable heterogeneity

c. Differences in cell dose and outcome assessment

d. Imprecision due to confidence intervals

**Supplementary Figure 15. GRADE assessment for Neurological Functional Recovery.**
